# Network Medicine‐Based Strategy Identifies Maprotiline as a Repurposable Drug by Inhibiting PD‐L1 Expression via Targeting SPOP in Cancer

**DOI:** 10.1002/advs.202410285

**Published:** 2024-11-05

**Authors:** Saisai Tian, Mengting Xu, Xiangxin Geng, Jiansong Fang, Hanchen Xu, Xinying Xue, Hongmei Hu, Qing Zhang, Dianping Yu, Mengmeng Guo, Hongwei Zhang, Jinyuan Lu, Chengyang Guo, Qun Wang, Sanhong Liu, Weidong Zhang

**Affiliations:** ^1^ Department of Phytochemistry School of Pharmacy Second Military Medical University Shanghai 200433 China; ^2^ Shanghai Frontiers Science Center of TCM Chemical Biology Institute of Interdisciplinary Integrative Medicine Research Shanghai University of Traditional Chinese Medicine Shanghai 201203 China; ^3^ Science and Technology Innovation Center Guangzhou University of Chinese Medicine Guangzhou 510006 China; ^4^ Institute of Digestive Diseases Longhua Hospital Shanghai University of Traditional Chinese Medicine Shanghai 200032 China; ^5^ Department of Respiratory and Critical Care Emergency and Critical Care Medical Center Beijing Shijitan Hospital Capital Medical University Beijing 100038 China; ^6^ State Key Laboratory for Quality Ensurance and Sustainable Use of Dao‐di Herbs Institute of Medicinal Plant Development Chinese Academy of Medical Sciences and Peking Union Medical College Beijing 100193 China; ^7^ The Research Center for Traditional Chinese Medicine Shanghai Institute of Infectious Diseases and Biosafety Institute of Interdisciplinary Integrative Medicine Research Shanghai University of Traditional Chinese Medicine Shanghai 201203 China

**Keywords:** colorectal cancer, immune checkpoint inhibitors, lung cancer, maprotiline, Mnet‐DRI, PD‐L1

## Abstract

Immune checkpoint inhibitors (ICIs) are drugs that inhibit immune checkpoint (ICP) molecules to restore the antitumor activity of immune cells and eliminate tumor cells. Due to the limitations and certain side effects of current ICIs, such as programmed death protein‐1, programmed cell death‐ligand 1, and cytotoxic T lymphocyte‐associated antigen 4 (CTLA4) antibodies, there is an urgent need to find new drugs with ICP inhibitory effects. In this study, a network‐based computational framework called **m**ulti‐**net**work algorithm‐driven **d**rug **r**epositioning targeting **I**CP (**Mnet‐DRI**) is developed to accurately repurpose novel ICIs from ≈3000 Food and Drug Administration‐approved or investigational drugs. By applying **Mnet‐DRI** to PD‐L1, maprotiline (MAP), an antidepressant drug is repurposed, as a potential PD‐L1 modifier for colorectal and lung cancers. Experimental validation revealed that MAP reduced PD‐L1 expression by targeting E3 ubiquitin ligase speckle‐type zinc finger structural protein (SPOP), and the combination of MAP and anti‐CTLA4 in vivo significantly enhanced the antitumor effect, providing a new alternative for the clinical treatment of colorectal and lung cancer.

## Graphical abstract

A network‐based computational framework known as Mnet‐DRI is developed to accurately identify maprotiline, an antidepressant, as a potential PD‐L1 inhibitor for use in colorectal and lung cancers. A series of experiments are conducted to demonstrate that maprotiline targets SPOP, resulting in the degradation of PD‐L1 and the activation of T cells, thereby suppressing the progression of colon and lung cancers.

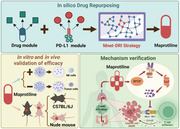

## 1 Introduction

Recently, tumor immunotherapy has become a focus in cancer treatment and has achieved a series of breakthroughs.^[^ ^1^ ^]^ Unlike traditional cancer treatment methods, such as radiotherapy and chemotherapy, immunotherapy aims to stimulate or enhance the immune system's ability to recognize and destroy cancer cells. Immunotherapy comprises various cancer therapies, including cell‐based therapies, immune checkpoint inhibitors (ICIs), cancer vaccines, and oncolytic viruses. ICIs are effective immunotherapies that can activate immune checkpoint (ICP) molecules, including programmed death protein‐1 (PD‐1) and cytotoxic T lymphocyte‐associated antigen 4 (CTLA4)‐related signaling pathways, thereby blocking the inflammatory response. Briefly, tumor cells achieve immune escape by activating signaling pathways associated with ICP molecules. ICIs can activate T lymphocytes and enhance tumor cell clearance.

Currently, the main ICP molecules include PD‐1, programmed cell death‐ligand 1 (PD‐L1), CTLA4, Siglec‐10, and Cluster of differentiation 24 (CD24). There is no doubt that labor‐based screening is unsuitable for identifying candidates specifically targeting ICP‐related molecules, as it is a time‐consuming, costly process with limited efficacy.^[^ ^2^, ^3^ ^]^ Several computational approaches have been proposed for screening immunotherapy candidate compounds.^[^ ^4^, ^5^, ^6^ ^]^ For example, Wu et al. identified small‐molecule compounds for anti‐PD‐1 immunotherapy via global gene network association.^[^ ^4^ ^]^ Wang et al. used a tumor immunological signature‐based computational method to identify novel immunotherapeutic compounds.^[^ ^6^ ^]^ However, the accuracy of these approaches is poor due to the lack of deep understanding of the pathological processes underlying ICP molecule‐associated biological networks.

Network‐based drug repurposing is a powerful strategy for identifying new therapeutic applications of approved drugs and could reduce the time and cost of drug development.^[^ ^7^, ^8^ ^]^ Genes that share analogous phenotypic functions often exhibit colocalization within a defined sector of protein‐protein interaction (PPI) network and can form disease modules.^[^ ^9^, ^10^ ^]^ Based on the drug targets and disease modules, network‐based drug repurposing can effectively screen potential approved drugs for multiple complex diseases. For example, Misselbeck et al. identified Bruton's tyrosine kinase (BTK) inhibitor ibrutinib as a prospective drug candidate for reducing chronic inflammatory conditions linked to obesity.^[^ ^11^ ^]^ Jessica C et al. identified metformin as a repurposable drug for treating atrial fibrillation.^[^ ^12^ ^]^ Fang et al. identified sildenafil as a candidate drug for Alzheimer's disease.^[^ ^13^ ^]^ However, the usefulness of the network‐based approach has not yet been fully exploited for drug repositioning targeting ICP molecules in human cancer.

In this study, we developed a network‐based computational framework called **Mnet‐DRI** to accurately repurpose ICIs from approved drugs by integrating PageRank, network proximity, functional similarity, and the RWR‐based network diffusion algorithm. We applied the **Mnet‐DRI** framework to PD‐L1 and identified maprotiline (MAP), a tetracyclic antidepressant that acts as a non‐selective monoamine reuptake inhibitor (NSRI) to alleviate depressive symptoms,^[^ ^14^ ^]^ as a candidate drug that inhibits PD‐L1 expression by targeting the SPOP. We found that combining MAP and anti‐CTLA4 antibodies could notably augment antitumor efficacy in patients with colorectal and lung cancers. The **Mnet‐DRI** framework can also repurpose in silico drugs by targeting other immunotherapeutic targets (PD‐L1, CTLA4, Siglec‐10, and CD24). We believe this approach can minimize the translational gap between genomic studies and drug development, a significant bottleneck in precision medicine.

## 2 Results

### 2.1 Computational Framework of Mnet‐DRI

**Figure 1:**
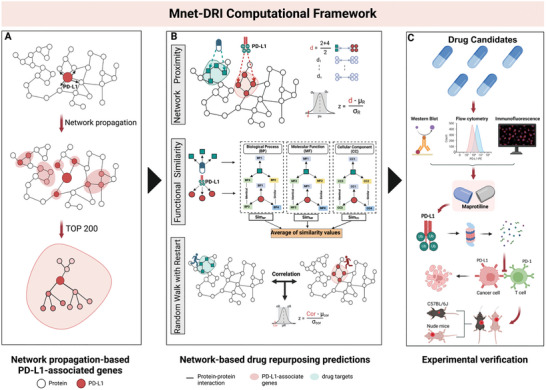
Computational framework of Mnet‐DRI. A) Network visualization to identify PD‐L1‐associated gene modules in a protein–protein interaction (PPI) network. B) Multiple network algorithms, including network proximity, functional similarity, and RWR‐based network diffusion algorithms, for repurposing ICIs from approved drugs. C) Experimental study to validate MAP as a repurposable drug for inhibiting PD‐L1 expression by targeting SPOP in colorectal and lung cancer. Created with BioRender.com.

To obtain a systems pharmacology perspective for identifying putative repurposed drugs targeting PD‐L1, we devised a network‐based computational framework, **Mnet‐DRI**, for in silico drug repurposing. The analytical procedure encompasses three interconnected parts, as depicted in **Figure** **1**. 1) The PD‐L1‐associated gene module was constructed by applying the PageRank algorithm to the PPI network. 2) Unique PD‐L1‐associated gene modules generated in the first step were targeted for in silico  drug repurposing, and three network‐based repurposing methodologies were employed. 2.1.) The network proximity (NP) algorithm ranks drugs based on the distance between the PD‐L1‐associated genes and protein targets of the drugs. 2.2.) The functional similarity (FS) algorithm ranks drugs based on the functional similarity between PD‐L1‐associated genes and protein targets of drugs. 2.3.) The RWR‐based network diffusion algorithm ranks drugs based on network similarity between PD‐L1‐associated genes and the protein targets of drugs. 3) Experimental validation of nominated drug candidates and mechanistic observations. Our in silico drug repurposing approach applies quality control steps for identifying drug candidates. We posited that only drugs predicted by all three methods were considered candidates. Finally, we identified MAP as a repurposable drug and extensive biological experiments were conducted to confirm its plausibility.

### 2.2 PD‐L1‐Associated Network Module Identified by Mnet‐DRI

**Figure 2:**
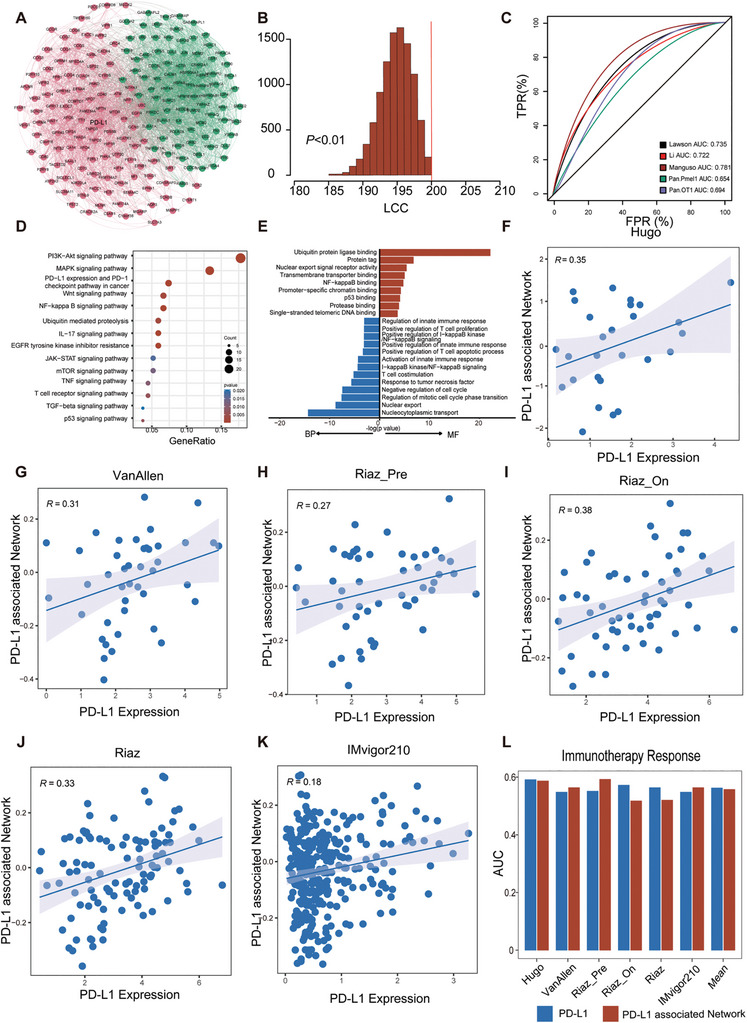
Identification of the PD‐L1‐associated gene module. A) Network visualization of the PD‐L1‐associated gene module using Gephi. The node color corresponds to the community membership of each node according to the modularity class in Gephi. Larger sizes highlight higher influence scores. B) Statistical analysis of the localization of the largest connected component (LCC) in the PD‐L1‐associated gene module. C) Evaluation of the PD‐L1‐associated gene module using benchmark gene sets and known pathways associated with immunotherapy. D) KEGG pathway annotation based on the PD‐L1‐associated gene module. E) GO annotation based on the PD‐L1‐associated gene module. F–K) The relationship between PD‐L1 expression and PD‐L1‐associated gene module in Hugo cohort, VanAllen cohort, Riaz cohort, and IMvigor210 cohort. “Pre” indicates before treatment; “on”, during treatment. L) The predictive capacity of immunotherapy response of PD‐L1 and PD‐L1‐associated gene modules.

To identify testable PD‐L1‐associated network modules in the human PPI network, we employed the PageRank network propagation algorithm to determine the influence of PD‐L1 target throughout the network. An inherent feature of network propagation is that nodes in proximity to the PD‐L1 target exhibit high influence scores. Furthermore, we identified genes with elevated influence scores (the top 200 genes) as constituents of the PD‐L1‐associated gene module (**Figure** **2**A and Table S1, Supporting Information). Notably, our prediction list included several genes involved in regulating PD‐L1, including IFI16, ATR, THADA, MYC, TP53, NPMI, and SMAD2, positively correlated with PD‐L1.^[^ ^15^, ^16^, ^17^, ^18^, ^19^, ^20^, ^21^ ^]^ CUL3 and ATM negatively regulate PD‐L1 in conjunction.^[^ ^22^, ^23^ ^]^ Coexpression of EGFR and PD‐L1 is associated with a poorer prognosis in patients.^[^ ^24^, ^25^ ^]^ Furthermore, our prediction list included genes, such as MDM2, NF1, and SOX2, associated with anti‐PD‐1 antibody therapy.^[^ ^26^, ^27^, ^28^ ^]^ Moreover, all genes formed a fully connected network, including 200 unique nodes and 3240 edges, significantly localized in the human interactome (*P* < 0.01; Figure 2B and Table S2, Supporting Information).

Numerous recent studies have identified critical genes linked to the interaction between cancer and T cells. We gathered five gene sets from these studies to comprehensively assess our predictions. Figure 2C displays the receiver operating characteristic curves and corresponding area under the curve (AUC) values for these five gene sets. The notably high AUC values signify the robust accuracy of our prediction method, indicating that a substantial portion of the genes in our association prediction list exhibited strong connections with response to anti‐PD‐L1 therapy. Next, we applied functional annotation to the PD‐L1‐associated gene module for association prediction. Kyoto encyclopedia of genes and genomes pathway enrichment revealed that many pathways known to be associated with the response to anti‐PD‐L1 therapy, such as the WNT pathway,^[^ ^29^ ^]^ PI3K‐AKT pathway,^[^ ^30^ ^]^ PD‐L1 expression and PD‐1 checkpoint pathway in cancer,^[^ ^31^ ^]^ MAPK pathway,^[^ ^32^ ^]^ NF‐kappa B signaling pathway,^[^ ^33^ ^]^ and TP53 pathway,^[^ ^16^ ^]^ were significantly enriched (Figure 2D).

Similarly, Gene Ontology (GO) enrichment analysis revealed several biological processes associated with the PD‐L1‐associated gene module, including positive regulation of the T cell apoptotic process,^[^ ^34^ ^]^ positive regulation of the innate immune response,^[^ ^35^ ^]^ and positive regulation of T cell proliferation (Figure 2E).^[^ ^36^, ^37^ ^]^ Furthermore, the PD‐L1‐associated network score for each patient was assessed using a gene set variation analysis. As demonstrated in Figures 2F–K, we observed a positive association between the expression of PD‐L1 and PD‐L1 network module scores in four immunotherapy cohorts, including three melanoma cohorts (Hugo et al.,^[^ ^38^ ^]^ VanAllen et al.,^[^ ^39^ ^]^ and Riaz et al.^[^ ^40^ ^]^) and one bladder cancer cohort (IMvigor210^[^ ^41^ ^]^). Next, we compared the predictive immunotherapy response performance of our PD‐L1‐associated network module and observed that the predictions of the PD‐L1‐associated network module were similar to or better than those of PD‐L1 expression (Figure 2L). Therefore, these results indicate the accuracy of our PD‐L1‐associated network module, which could be used for subsequent analyses.

### 2.3 In Silico Screening of Drug Candidates by Mnet‐DRI

To identify potential PD‐L1 inhibitors, we employed three network‐repurposing methodologies, NP, FS, and RWR, to predict the expected efficacy of 2937 drugs. All pipelines depend on the same input data to ensure consistency and maintain their prospective nature, with all subsequent analyses building on the foundation of this initial prediction list.

#### 2.3.1 NP Method

**Figure 3:**
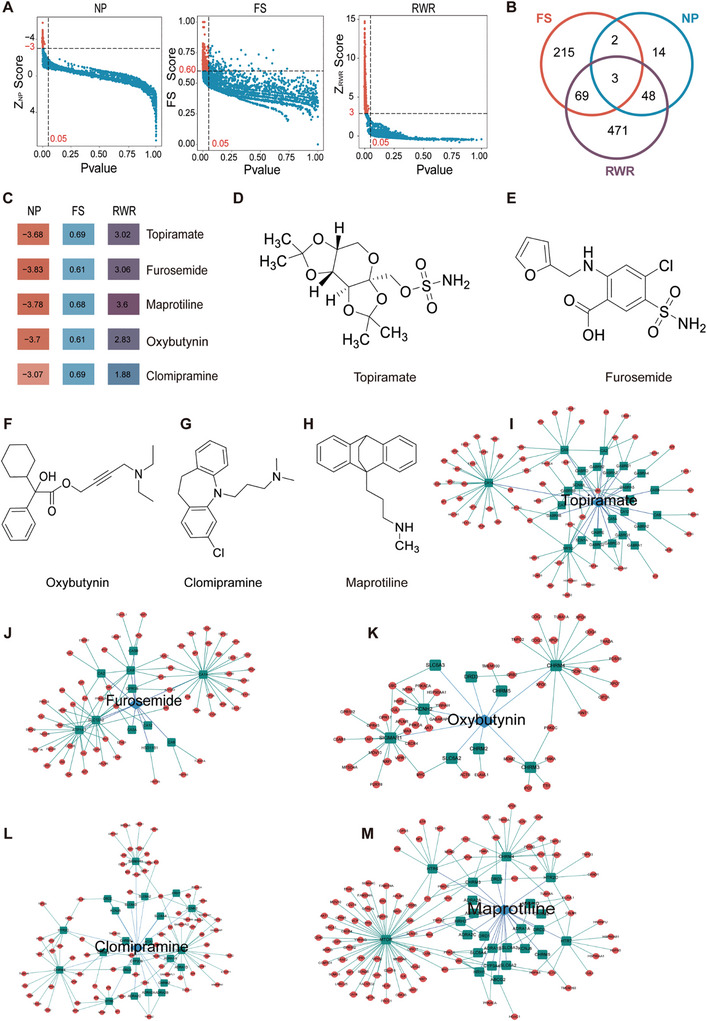
Drug repurposing using Mnet‐DRI. A) Computational results based on the multiple network approach. Candidates are labeled in tangerine. B) Candidate overlap of three network algorithms. C) Visualization of candidate scores using different methods. The darker the color is, the higher the score. D–H) Chemical structures of the candidates. I–M) A subnetwork highlighting the candidate drugs associated with the PD‐L1‐associated gene module and their associated targets.

The network proximity between drug targets and the PD‐L1‐associated module was calculated using Equations (2), (3), (4). Furthermore, we calculated the Z_NP_ score and performed 1000 permutation tests to quantify the significance of the network proximity between drug targets and proteins in the PD‐L1‐associated module within the human interactome network. A higher network proximity (quantified by a lower Z_NP_ score) represents a strong network relationship. Using the cutoff values of Z_NP_ < −3 and *P* < 0.05, we focused on 67 drug candidates (**Figure** **3**A and Table S3, Supporting Information).

#### 2.3.2 FS Method

To assess the functional similarity between PD‐L1‐associated genes and drug targets, GO annotations, including biological process (BP), molecular function (MF), and cellular component (CC), were used to calculate a mean similarity score ranging between 0 and 1 using Equations 5 and 6, in which drugs with high similarity scores (close to 1) indicated greater functional similarity. Moreover, 1000 permutation tests were performed to quantify the significance of functional similarity. Using the cutoff values of FS > 0.6 and *P* < 0.05, we identified 289 drug candidates (Figure 3A and Table S4, Supporting Information).

#### 2.3.3 RWR Method

RWR‐based network diffusion methodology was used to evaluate the efficacy of the drug using Equations (7), (8), (9). Moreover, we calculated the Z_RWR_ score and performed 1000 permutation tests to quantify the significance of the RWR between drug targets and the PD‐L1‐associated gene module in the human interactome network. A higher Z_RWR_ represents a strong network relationship. Using cutoff values of Z_RWR_ > 3 and *P* < 0.05, we focused on 591 drug candidates (Figure 3A and Table S5, Supporting Information).

Because the various pipelines successfully predicted distinct subsets of drugs, we identified three drugs (topiramate, furosemide, and maprotiline [MAP]) for which all pipelines provide predictions. Moreover, given that oxybutynin and clomipramine have potential antitumor activity^[^ ^42^, ^43^ ^]^ and overlap between the NP and FS methods, they were included in subsequent experiments, and we performed biological validation of these five drugs (Figures 3B,C). The five drugs had diverse chemical structures and pharmacological categories (Figure 3D–H). Topiramate, an anticonvulsant drug used to control epilepsy and prophylactically,^[^ ^44^ ^]^ targeted 58 PD‐L1‐associated module genes (Figure 3I). Furosemide, a Food and Drug Administration (FDA)‐approved diuretic for treating hypertension and edema in congestive heart failure, liver cirrhosis, renal disease, and hypertension,^[^ ^45^, ^46^ ^]^ has been revealed to target 62 PD‐L1‐associated module genes (Figure 3J). Oxybutynin is an antimuscarinic agent that reduces detrusor muscle activity,^[^ ^47^, ^48^ ^]^ relaxing the bladder and preventing the urge to void (Figure 3K). Clomipramine and MAP are antidepressants used to treat depressive illness, and their effects on these patients are illustrated in Figure 3L,M.^[^ ^49^, ^50^, ^51^, ^52^ ^]^

### 2.4 Identification of MAP Hydrochloride as a New Candidate Drug

**Figure 4:**
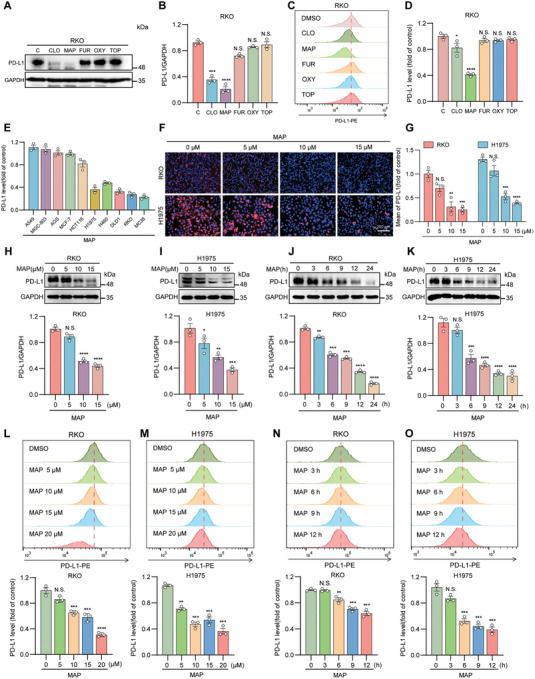
MAP negatively regulates PD‐L1 expression in cancer cells. A,B) Verification of five compounds that may negatively regulate PD‐L1 in RKO cells with high PD‐L1 expression by protein immunoblotting (A). (B) Quantitative results of (A). C,D) Detection of the effects of five compounds on membrane PD‐L1 in RKO cells by flow cytometry (C). (D) Quantitative results of (C). E) Quantitative graph of the downregulation of PD‐L1 on different tumor cells by flow cytometry with MAP. F,G) Expression of membrane PD‐L1 detected by immunofluorescence after treatment with MAP (10 µm) for 24 h in RKO and H1975 cells. DAPI staining in red indicates PD‐L1, blue indicates the cell nucleus and the scale bar represents 200 µm. (G) Fluorescence quantification results of (F). H,I) Western blotting was used to detect PD‐L1 levels in RKO cells (H) and H1975 cells (I) after treatment with different concentrations of MAP for 24 h. J,K) Protein blotting analysis of PD‐L1 levels in RKO cells (J) and H1975 cells (K) after treatment with 10 µm MAP for different durations. L,M) Detection of membrane PD‐L1 levels in RKO cells (L) and H1975 cells (M) after treatment with different concentrations of MAP for 24 h by flow cytometry. N,O) Analysis of membrane PD‐L1 levels in RKO cells (N) and H1975 cells (O) after treatment with 10 µm MAP for different durations by flow cytometry. The data shown are the mean value ± standard error of the mean (SEM). Statistical differences were determined by Student's t‐test. *p < 0.05; **p < 0.01; ^***^ *p* < 0.001; ^****^ *p* < 0.0001; N.S. not significant.

Flow cytometry and Western blotting were used to validate the effects of the five drugs on RKO cells characterized by elevated PD‐L1 expression. Our results indicated that MAP and clomipramine hydrochloride (CLO) decreased the levels of PD‐L1 in RKO cells, with MAP demonstrating particularly significant effects. (**Figure** **4**A–D). CLO has been reported in the literature,^[^ ^42^ ^]^ and its effect on downregulating PD‐L1 is not as significant as that of MAP; therefore, our subsequent experiments focused mainly on in‐depth research on MAP. We further studied the ability of MAP to downregulate PD‐L1 expression on the cell membrane in different cell lines using flow cytometry. The results exhibited that MAP had a stronger downregulatory effect on PD‐L1 on colorectal and lung cancer cell membranes than on other cell lines (Figure 4E). Subsequently, using the cell counting kit‐8 (CCK‐8) and EdU experiments, we determined that 10 µm was a safe and effective concentration of MAP (Figure S1A–E, Supporting Information). Consequently, this concentration was used in subsequent experiments on RKO and H1975 cells. Immunofluorescence experiments confirmed that MAP could reduce the transport of PD‐L1 to the plasma membrane in RKO and H1975 cells in a time‐ and concentration‐dependent manner (Figure 4F,G; Figure S2A–D, Supporting Information). Similar decreases in PD‐L1 protein expression were observed in RKO, H1975, and MC38 cells (Figure 4H–K; Figure S2E–H, Supporting Information). Moreover, MAP exhibited this characteristic inhibitory effect on the levels of PD‐L1 on the cell membranes of RKO and H1975 cells; it also had a similar effect on MC38, HT29, and DLD1 cells (Figure 4L–O; Figure S2I–P, Supporting Information). These results indicate that MAP can reduce PD‐L1 expression in colorectal and lung cancer cells.

### 2.5 MAP Promoted T Cell Activation and T Cell‐Mediated Killing of Cancer Cells In Vitro and In Vivo

To further assess the in vitro and in vivo antitumor efficacy of MAP, we performed co‐culture experiments with RKO or H1975 cells and Jurkat cells^[^ ^53^ ^]^ and evaluated the survival of tumor cells using crystal violet staining. MAP reduced the survival rate of tumor cells by enhancing the cytotoxicity of T cells (Figure S3A–D, Supporting Information). The antitumor effect of MAP on MC38 tumor growth was examined by the oral administration of corn oil or MAP once daily for 16 days. MAP significantly suppressed MC38 tumors at 10, 20, and 40 mg k^−1^g^−1^ with inhibition rates of 22.19, 53.11, and 70.16%, respectively. This result was further confirmed by comparing tumor weights (Figure S4A–D, Supporting Information). Moreover, the inhibitory effect of MAP on MC38 tumors and Lewis's tumors was abolished in immunocompromised nude mice, which suggests that the antitumor effect of MAP can be attributed to its stimulation of the T cell‐mediated immune response (Figure S6A–C,E–G, Supporting Information). Moreover, by comparing the body weight changes and histological staining results of the major organs in the different groups of mice, it was determined that MAP had a non‐significant toxic effect on mice at this dosage (Figures S4E, S5, S6D,H–J, Supporting Information).

In the tumor microenvironment, myeloid‐derived suppressor cells (MDSCs) and regulatory T cells (Tregs) promote tumor immune escape through the release of immunosuppressive factors, while activated MDSCs and Tregs express large amounts of PD‐L1, which interacts with PD‐1 on T cells and can also lead to T cell exhaustion.^[^ ^54^, ^55^ ^]^ Next, we performed a flow cytometric analysis of tumor tissue. We found that the numbers of activated MDSCs (CD11b^+^Gr‐1^+^) and Tregs (CD4^+^CD25^+^Foxp3^+^) among the tumor‐infiltrating lymphocytes in the MAP treatment group were significantly reduced (Figure S4F–I, Supporting Information). Additionally, flow cytometric analysis revealed that the levels of granzyme B, an indicator of cytotoxic T‐cell activation, increased in a dose‐dependent manner with MAP, indicating that MAP can stimulate the activity of cytotoxic T lymphocytes (Figure S4J,K, Supporting Information). Furthermore, immunohistochemical analysis revealed that MAP dose‐dependently increased the levels of CD3 (a marker of T cells), CD4 (regulatory T cells), CD8 (cytotoxic T cells), and C‐caspase‐3 (mitotic caspase‐3) in tumors. Conversely, the levels of Ki‐67 (a marker of proliferation), PD‐L1, and Foxp3 (an immune suppressor molecule) were significantly decreased (Figure S4L,M, Supporting Information). Our data depicted that MAP may exert significant antitumor effects by activating tumor‐infiltrating T cells.

### 2.6 MAP Promoted the Ubiquitination of PD‐L1 Protein to Facilitate its Degradation

**Figure 5:**
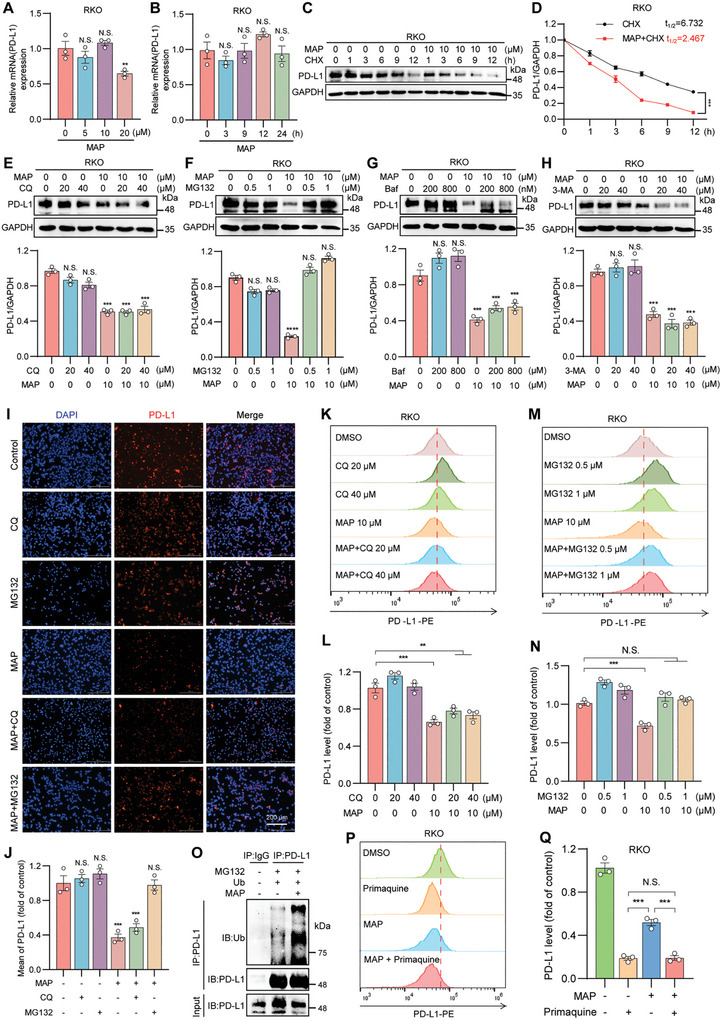
MAP downregulates PD‐L1 through the proteasome pathway. A,B) RT‐PCR analysis of PD‐L1 levels in RKO cells treated with different concentrations of MAP for 24 h (A) or treated with 10 µm MAP for different durations (B). C) Western blotting detection of PD‐L1 expression in RKO cells treated with DMSO or 10 µm MAP for specified durations in the presence of CHX (50 mg mL^−1^) (C). D) Quantification of (C). E–H) Western blotting detection of PD‐L1 expression in RKO cells treated with MAP in combination with chloroquine (E), proteasome inhibitor MG132 (F), lysosome inhibitor bafilomycin (G), or autophagy inhibitor 3‐methyladenine (H). I) DAPI staining of PD‐L1 on the membrane of RKO cells treated with MAP (10 µm) in combination with MG132 (5 µm) or chloroquine (40 µm) for 12 h. The scale bar represents 200 µm. J) Quantification of (I). K–N) Flow cytometry analysis was used to determine whether lysosome inhibitor CQ (K) and proteasome inhibitor MG132 (M) affect the downregulation of PD‐L1 by MAP in RKO cells. The quantified results of (L) and (N) are shown in (K) and (M), respectively. O) Overexpression of Ub in RKO cells followed by immunoprecipitation to detect MAP‐induced ubiquitination of PD‐L1. Immunoprecipitation of ubiquitinated PD‐L1 protein using Flag beads and immunoblotting with a Ub antibody. P,Q) Flow cytometry analysis of PD‐L1 levels on the membrane of RKO cells treated with MAP in combination with the endocytosis inhibitor primaquine (P); (Q) shows the quantitative results of (P). The data shown are the mean value ± standard error of the mean (SEM). Statistical differences were determined by Student's t‐test. ^*^ *p* < 0.05; ^**^ *p* < 0.01; ^***^ *p* < 0.001; ^****^ *p* < 0.0001; N.S. not significant.

Given that PD‐L1 downregulation by MAP is apparent, we explored its degradation mechanism in the subsequent experiments. RT‐PCR revealed that MAP had a non‐significant effect on the mRNA level of PD‐L1 in RKO cells at either time or concentration (**Figure** **5**A,B). To further confirm that the degradation of PD‐L1 by MAP results from post‐translational regulation, RKO cells were treated with the protein synthesis inhibitor cycloheximide (CHX) to determine the half‐life of PD‐L1. As revealed in Figure 5C,D, PD‐L1 degradation was higher in MAP‐treated cells than in untreated cells in CHX. These findings indicate that PD‐L1 downregulation triggered by MAP occurs primarily at the protein level and does not affect its transcription.

The expression and regulation of PD‐L1 involves various cellular biological processes, including ubiquitination and lysosomal degradation.^[^ ^56^ ^]^ These processes are crucial for maintaining immune system homeostasis and evasion of tumor immune surveillance. To elucidate the specific pathway by which MAP participates in PD‐L1 degradation, we conducted cotreatment experiments with MAP and various inhibitors, including MG132 (a proteasome inhibitor), bafilomycin (Baf, a lysosome inhibitor), chloroquine (CQ, a lysosome inhibitor), and 3‐methyladenine (3‐MA, an autophagy inhibitor), in RKO cells. Our findings revealed that the destabilization of PD‐L1 caused by MAP could be rescued by MG132 but not by Baf, CQ, or 3‐MA (Figures 5E–H and S7A, Supporting Information). Consistent with Western blotting results, immunofluorescence, and flow cytometry results revealed that PD‐L1 degradation on cell membranes could also be reversed by proteasomal inhibitors (Figure 5I–N). To validate the ex vivo effect of MAP, we analyzed PD‐L1 ubiquitination levels in MC38 subcutaneous tumors post‐treatment and found a significant increase in ubiquitination, accompanied by a marked reduction in PD‐L1 expression in tumor tissues of the MAP‐treated group (Figure S7B, Supporting Information). Furthermore, we conducted immunoprecipitation experiments to detect the ubiquitination of PD‐L1 in the presence of MAP and found that MAP significantly triggered PD‐L1 ubiquitination (Figure 5O).

Moreover, many studies have revealed the significance of endocytic recycling in sustaining PD‐L1 protein.^[^ ^57^, ^58^, ^59^ ^]^ To explore whether MAP induces PD‐L1 degradation by interfering with this physiological process, we used primaquine, an inhibitor of endocytic recycling, to prevent PD‐L1 recycling to the plasma membrane, as described in previous literature.^[^ ^60^ ^]^ As anticipated, primaquine swiftly depleted PD‐L1 on the cell membrane, indicating that a significant portion of surface PD‐L1 was consistently internalized and recycled. Notably, MAP did not induce any further loss of PD‐L1 or accelerate its degradation in the presence of primaquine (Figure 5P,Q). These findings indicate that MAP inhibits PD‐L1 cycling in the plasma membrane.

### 2.7 MAP Promoted PD‐L1 Degradation by Targeting SPOP

**Figure 6:**
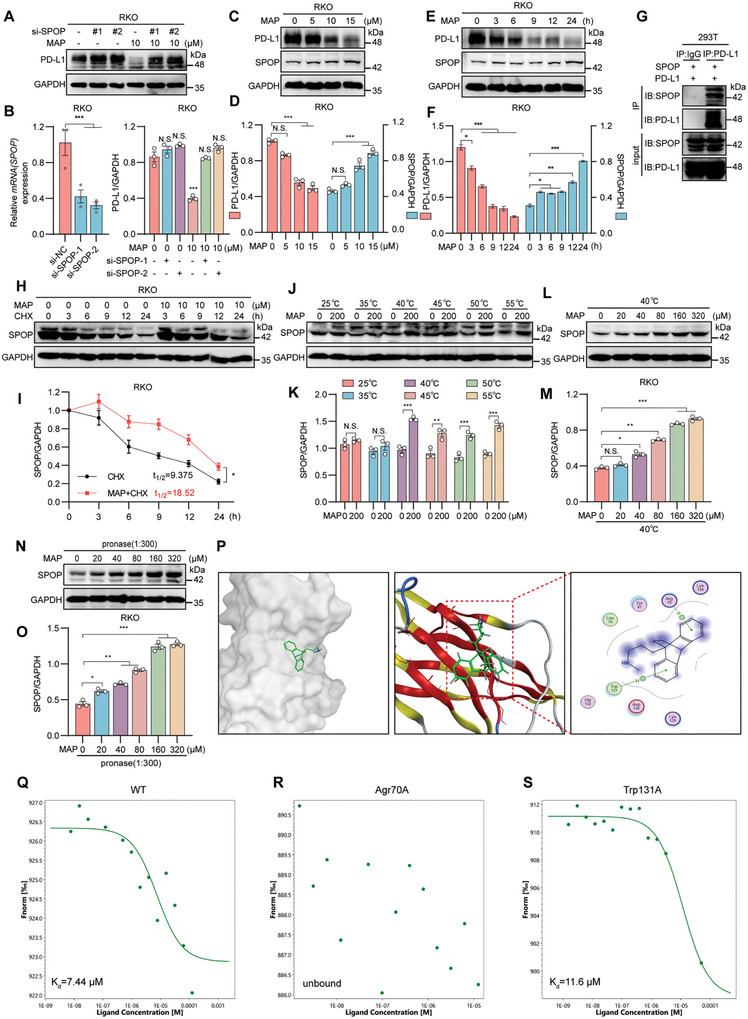
SPOP targeted to MAP promotes the degradation of PD‐L1. A) After treatment with siRNA targeting SPOP or negative control siRNA (siRNA‐NC), Western blotting was performed to evaluate the protein expression of PD‐L1 in RKO cells treated with MAP. After SPOP silencing, the addition of MAP did not decrease PD‐L1 expression. B) Knockdown efficiency of SPOP detected by RT‐qPCR and the quantitative results for (A). C–F) Western blotting was utilized to assess the overall protein levels of SPOP following treatment of RKO cells with varying concentrations of MAP for 24 h (C) or exposure of RKO cells to 10 µm MAP for different durations (E), (D) and (F) are quantifications of (C) and (E), respectively. G) 293T cells transfected with PD‐L1 plasmid and SPOP plasmid were collected for immunoprecipitation (IP) and IB analysis. H,I) The protein level of SPOP in RKO cells treated with 10 µm MAP and cycloheximide (CHX) (50 mg mL^−1^) was detected by western blotting (H). (I) Quantification of the data in (G). J,K) The thermal stability of SPOP during interactions with MAP at 25, 35, 40, 45, 50 and 55°C was determined by the CETSA method. (K) Quantitative analysis of the results in (J). L,M) Stability of SPOP at 40°C under different concentrations of MAP (L); (M) is the quantitative result of (L). N,O) Stability of SPOP at various MAP concentrations with a 1:300 pronase‐to‐protein ratio; (O) quantification (N). P) Molecular docking of MAP with SPOP. Q) Wild‐type SPOP binds to MAP. R,S) MAP binds to SPOP mutated at Agr70 (R) or Trp131 (S). The data shown are the mean ± standard error of the mean (SEM). Statistical differences were determined by Student's t‐test. ^*^ *p* < 0.05; ^**^ *p* < 0.01; ^***^ *p* < 0.001; ^****^ *p* < 0.0001; N.S., not significant.

Ubiquitin ligases and deubiquitinases (DUBs) are vital for the ubiquitination of proteins,^[^ ^61^, ^62^ ^]^ among which the E3 ubiquitin ligase is a key enzyme in the ubiquitin‐protein linkage system responsible for linking small protein ubiquitins to target proteins, thereby regulating their function, localization, and degradation.^[^ ^63^ ^]^ Contrarily, deubiquitinating enzymes regulate protein stability and function by removing the ubiquitin chain and reversing the ubiquitination process.^[^ ^64^, ^65^ ^]^ E3 ligases such as HRD1, ARIH1, BTRC, A20, STUB1, MARCH8, and SPOP have been reported to ubiquitinate PD‐L1, facilitating its degradation.^[^ ^57^, ^66^, ^67^, ^68^, ^69^, ^70^, ^71^, ^72^ ^]^ Conversely, the deubiquitinating enzymes OTUB1, CSN5, and USP22 are recognized for stabilizing PD‐L1.^[^ ^73^, ^74^, ^75^ ^]^ With this in mind, we knocked down these E3 ligases using siRNA in RKO cells or overexpressed these DUBs using plasmids and found that the reduction in PD‐L1 levels induced by MAP could be counteracted solely by SPOP suppression (**Figure** **6**A,B; Figure S7D–L, Supporting Information), which led us to speculate that SPOP might be a potential target for MAP. Subsequent data corroborated this hypothesis, illustrating that MAP elevated SPOP protein levels in a dose‐ and concentration‐dependent manner (Figure 6C–F). Meanwhile, our experiments confirmed the interaction between SPOP and PD‐L1 (Figure 6G), aligning with findings from related studies.^[^ ^69^ ^]^

**Figure 7:**
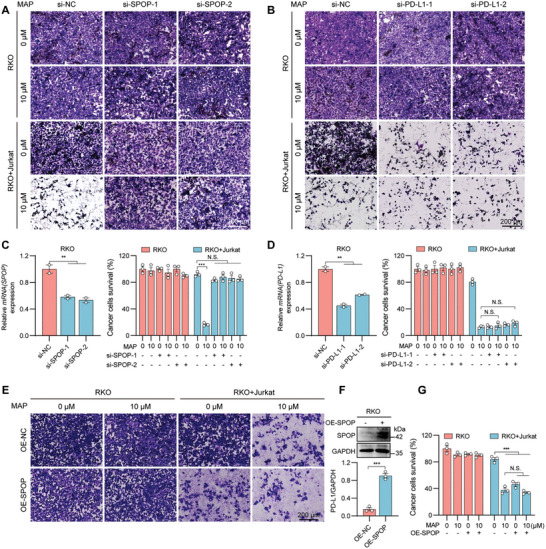
MAP enhances T cell killing in vitro by targeting SPOP to promote PD‐L1 degradation. A–D) Tumor cells that survived after knockdown of SPOP (A) or PD‐L1 (B) and were then cocultured with Jurkat cells for a period of time were observed by crystal violet staining, after which the results were quantified, and the knockdown efficiencies of SPOP (C) and PD‐L1 (D) were detected by RT‐qPCR. E–G) Tumor cells that survived co‐incubation with Jurkat cells after SPOP overexpression were analyzed using crystal violet staining (E), and SPOP overexpression efficiency was assessed via Western blotting (F). The quantitative data from (E) is shown in (G). The data shown are the mean ± standard error of the mean (SEM). Statistical differences were determined by Student's t‐test. ^*^ *p* < 0.05; ^**^ *p* < 0.01; ^***^ *p* < 0.001; ^****^ *p* < 0.0001; N.S., not significant.

Furthermore, CHX chase assays confirmed that MAP extended the half‐life of the SPOP protein (Figure 6H,I). Additionally, we conducted a network analysis to identify genes specifically expressed in the colon using data from the GTEx database, which yielded 153 genes with a Z‐score greater than 2.5 (Table S6, Supporting Information). As revealed in Figure S7C (Supporting Information), SPOP had shorter network distances to the PD‐L1‐associated module in the human interactome. These results suggest that SPOP is involved in regulating PD‐L1 by MAP. Subsequently, we performed a cellular thermal shift assay (CETSA) to test whether there was a direct binding interaction between MAP and SPOP. As demonstrated in Figure 6J–O, MAP significantly increased SPOP accumulation, and the stability of SPOP at 40°C and a streptavidin:protein lysate ratio of 1:300 increased with increasing MAP concentration. We performed docking simulations using the MOE software to identify specific amino acid residues in SPOP that interact with MAP (Figure 6P). The simulations indicated potential interactions between MAP and residues such as Arg70 and Trp131. We constructed GFP‐tagged wild‐type, Arg70, or Trp131 mutant SPOP plasmids and analyzed their direct binding using microthermophoresis (MST). The K_d_ value for MAP binding to the wild‐type SPOP was estimated to be 7.44 µm (Figure 6Q). Mutation of the Arg70 site abolished the binding interaction between MAP and SPOP (Figure 6R), whereas mutation of the Trp131 site did not affect the binding (Figure 6S). These results suggest that MAP binds to SPOP through the Arg70 site. Furthermore, MAP did not enhance T cell‐mediated killing when SPOP was knocked down in RKO cells (**Figure** **7**A,C), whereas overexpression of SPOP or MAP treatment enhanced T cell killing of tumor cells. However, overexpression of SPOP in combination with MAP did not further enhance T cell killing of tumor cells (Figure 7E–G), indicating that MAP mediates tumor cell killing via SPOP in vitro. In conclusion, MAP promotes PD‐L1 degradation by targeting SPOP, thereby exerting antitumor effects.

### 2.8 The Synergistic Effect of MAP and Anti‐CTLA4 in Colorectal and Lung Cancers

**Figure 8:**
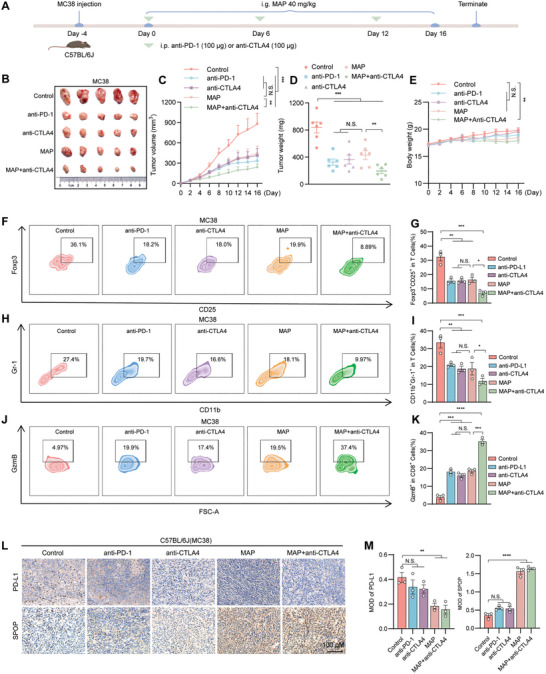
The combination of MAP and anti‐CTLA4 enhances the efficacy of treatment for colorectal cancer. A–E) C57BL/6J (female) mice were orally treated with corn oil, anti‐PD‐1, anti‐CTLA4, MAP (40 mg k^−1^g^−1^), or MAP (40 mg k^−1^g^−1^) combined with anti‐CTLA4 and were subcutaneously inoculated with MC38 colorectal cancer cells (8 × 10^5^ cells/mouse), n = 5 mice per group. (A) Scheme representing the experimental procedure. i.g., intragastric; i.p., peritoneal injection. (B) Representative solid tumors were excised from mice in different groups. (C) Tumor growth curves of mice receiving different treatments. (D) Tumor weights were excised from mice in each group that received different treatments. (E) Changes in the body weights of the mice in each group were recorded. F–K) Representative flow cytometry plots of CD4^+^CD25^+^Foxp3^+^ (F) and CD11b^+^Gr‐1^+^ (H) populations, as well as GzmB levels in CD3^+^CD8^+^TILs (J) from MC38 tumors treated with different therapies. (G), (I), and (K) are the respective quantitative results. L,M) Immunohistochemistry showing the expression of PD‐L1 and SPOP in tumor tissues from different groups of MC38 (L) mice; scale bar = 100 µm, (L) is the quantitative analysis of (M). The data shown are the mean ± standard error of the mean (SEM). Tumor growth data from the mice were analyzed by two‐way ANOVA with repeated measures. Statistical differences in other data were determined by Student's t‐test. ^*^ *p* < 0.05; ^**^ *p* < 0.01; ^***^ *p* < 0.001; N.S., not significant.

**Figure 9:**
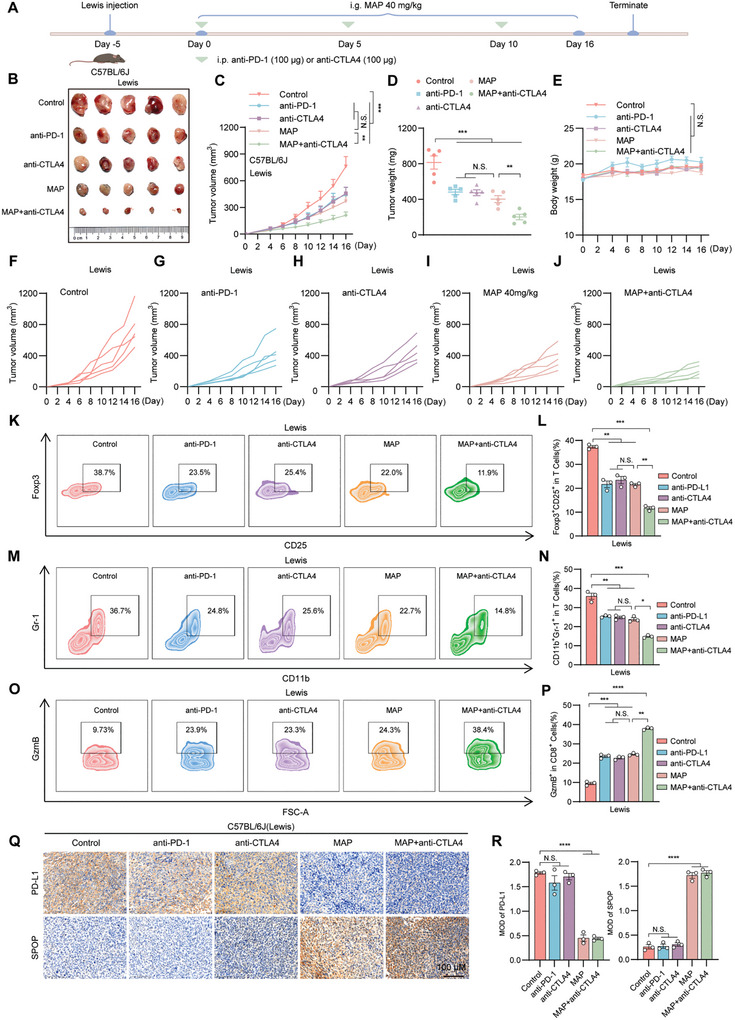
The combination of MAP and anti‐CTLA4 enhances the anti‐lung cancer effect. A–E) C57BL/6J (female) mice were orally treated with corn oil, anti‐PD‐1, anti‐CTLA4, MAP (40 mg k^−1^g^−1^), or MAP (40 mg k^−1^g^−1^) combined with anti‐CTLA4 and were subcutaneously inoculated with Lewis's lung cancer cells (3 × 10^7^ cells/mouse), n = 5 mice per group. (A) Scheme representing the experimental procedure. i.g., intragastric; i.p., peritoneal injection. (B) Representative solid tumors were excised from mice in different groups. (C) Tumor growth curves of mice receiving different treatments. (D) Tumor weights were excised from mice in each group that received different treatments. (E) Changes in the body weights of the mice in each group were recorded. F–J) Tumor volume growth curves for single mice in the control group (F), anti‐PD‐1 group (G), anti‐CTLA4 group (H), MAP group (I), and MAP combined with anti‐CTLA4 group (J). K–P) Representative flow cytometry plots of CD4^+^CD25^+^Foxp3^+^ (K) and CD11b^+^Gr‐1^+^ (M) populations, as well as GzmB levels in CD3^+^CD8^+^TILs (O) from Lewis tumors treated with different therapies. (L), (N, and (P) are the respective quantitative results. Q,R) Immunohistochemistry showing the expression of PD‐L1 and SPOP in tumor tissues from different groups of Lewis (O) mice; scale bar = 100 µm, (R) is the quantitative analysis of (Q), respectively. The data shown are the mean ± standard error of the mean (SEM). Tumor growth data from the mice were analyzed by two‐way ANOVA with repeated measures. Statistical differences in other data were determined by Student's t‐test. ^*^ *p* < 0.05; ^**^ *p* < 0.01; ^***^ *p* < 0.001; N.S., not significant.

In vitro experiments, we used siRNA to interfere with PD‐L1 expression in RKO cells and observed that this interference did not further enhance the ability of MAP to increase the Jurkat cell‐mediated killing of RKO cells. These findings confirmed that MAP enhanced T‐cell killing by reducing PD‐L1 expression in tumors (Figure 7B,D). Moreover, combination therapy involving anti‐PD‐1 or anti‐CTLA4 agents has substantially enhanced treatment response and survival rates in cancer patients.^[^ ^60^, ^76^, ^77^ ^]^ To investigate the potential synergistic effect of combining MAP and anti‐CTLA4 antibody therapy in the context of immunosuppression, we conducted an experiment in which female C57BL/6J mice were subcutaneously implanted with MC38 and Lewis cells. Mice bearing MC38 or Lewis tumors were treated with corn oil, MAP, anti‐CTLA‐4 antibody, anti‐PD‐1 antibody, or their combination. Our results demonstrated that, compared with MAP or anti‐CTLA4 therapy alone, combination therapy further inhibited the tumor growth rate and volume (**Figures** **8**A–E and **9**A–J). Consistent with the above results, flow cytometry analysis revealed that the combination therapy group exhibited the lowest levels of MDSCs (CD11b^+^Gr‐1^+^) and Tregs (CD4^+^CD25^+^Foxp3^+^) but the highest level of granzyme B (Figures 8F–K and 9K–P). Visceral analyses of mice subjected to hematoxylin‐eosin (HE) staining confirmed the safety of the drugs in each treatment group (Figure S8A,B, Supporting Information). Immunohistochemistry results demonstrated a significant decrease in PD‐L1 levels in tumor tissues following MAP treatment and a notable increase in SPOP levels (Figures 8L,M and 9Q,R). The combination of MAP and anti‐CTLA‐4 therapy effectively enhanced the infiltration of NK and T cells and promoted tumor cell apoptosis (Figures S9A,B and S10A,B, Supporting Information).

Further analysis of immune cell changes in the tumor microenvironment post‐MAP administration revealed increased CD11c^+^ dendritic cells (DCs), indicating improved antigen presentation. There was a significant increase in M1‐type macrophages (CD86^+^ F480^+^) and a decrease in M2‐type macrophages (CD206^+^), suggesting that MAP plays a role in reducing PD‐L1 levels, alleviating T‐cell immunosuppression, and inducing M1 macrophage polarization (Figures S9C,D and S10C,D, Supporting Information). Therefore, our findings suggest that MAP can transform the immune microenvironment from immunosuppressive to immune activation. When MAP is combined with anti‐CTLA4 therapy, it synergistically inhibits the proliferation of colorectal and lung cancer cells, thereby further promoting antitumor effects.

### 2.9 Association of SPOP with the Clinical Management of Lung and Colon Cancer

**Figure 10:**
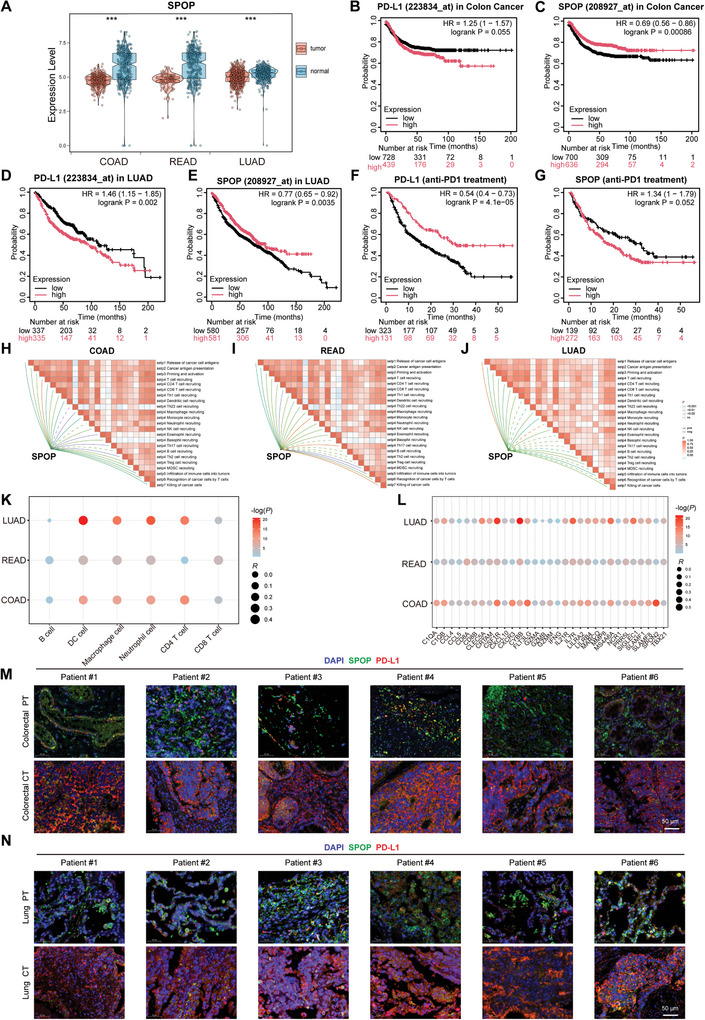
Clinical relevance of SPOP to the treatment of patients with lung and colon cancer. A) The expression of SPOP in cancer tissues and normal tissues, including TCGA‐COAD, TCGA‐READ, and TCGA‐LUAD data. B–G) The survival of cancer patients stratified by the expression of PD‐L1 or SPOP was compared by two‐sided log‐rank analysis in colon cancer, LUAD, and immunotherapy cohorts. H–J) Correlations between SPOP and the steps of the cancer immune cycle in COAD, READ, and LUAD. K) Correlations between SPOP and the infiltration levels of six tumor‐associated immune cell types according to the TIMER algorithm. L) Correlations between SPOP and the effector genes of the above tumor‐associated immune cells. M,N) Fluorescent double‐stained IHC was utilized to examine the levels of PD‐L1 and SPOP expression in paraneoplastic (PT) and cancerous (CT) tissues from patients with colorectal cancer (I) and lung cancer (J).

By mining TCGA and GTEx databases, we found lower expression levels of SPOP in COAD, READ, and LUAD tissues than in normal tissues (**Figure** **10**A,B). Moreover, in clinical treatment, we found that patient outcomes were negatively correlated with PD‐L1 and positively correlated with SPOP (Figure 10B–E). After anti‐PD‐1 antibody treatment, patients with high PD‐L1 and low SPOP expression had a better prognosis (Figure 10F,G). Consistent with our expectations, SPOP revealed a positive correlation with key steps of the cancer‐immunity cycle, such as the release of cancer cell antigens (Step 1), cancer antigen presentation (Step 2), trafficking of immune cells to tumors (Step 4) (i.e., CD4 T cell, CD8 T cell, macrophage, and monocyte recruitments) and infiltration of immune cells into tumors (Step 5) in COAD, READ, or LUAD (Figure 10H–J). Moreover, SPOP was positively correlated with CD8^+^ T cells, CD4^+^ T cells, B cells, and macrophages (Figure 10K) and correlated with effector genes of tumor‐infiltrating immune cells (Figure 10L). Furthermore, SPOP was positively correlated with most immunomodulators, such as CXCL12, CXCL16, CXCL10, CCL2, and others (Tables S7‐S9, Supporting Information). Additionally, we analyzed the expression levels of PD‐L1 and SPOP in both paracancerous and cancerous tissues of patients with colon and lung cancer. As illustrated in Figures 10M,N and S11A,B (Supporting Information), PD‐L1 expression was significantly higher in cancerous tissues than in paracancerous tissues, whereas SPOP expression was lower in cancerous tissues. Collectively, these findings suggest that SPOP downregulates PD‐L1 expression in tumor tissues.

## 3 Discussion

The FDA in the United States has approved Ipilimumab, an antibody that targets cytotoxic T‐lymphocyte‐associated antigen 4 (CTLA‐4)^[^ ^78^ ^]^ along with PD‐1/PD‐L1 pathway antibodies like pembrolizumab (PD‐1)^[^ ^79^ ^]^ and atezolizumab (PD‐L1).^[^ ^80^ ^]^ These ICIs have demonstrated effectiveness in various malignancies through immune checkpoint blockade.^[^ ^81^ ^]^ These monoclonal antibodies have exhibited excellent clinical efficacy in numerous patients, revolutionizing therapeutic approaches for multiple tumors. Nevertheless, most patients with tumors do not benefit from these antibody treatments. Presently, there are certain limitations to monoclonal antibody therapy, including the absence of sensitive predictive biomarkers for clinical application, a low overall patient response rate to antibody therapy, the inadequacy of existing efficacy assessment indicators for objective evaluation, and the manifestation of potent adverse effects associated with tumor immunotherapy.^[^ ^82^ ^]^ Small‐molecule inhibitors are considered promising alternative strategies because they can traverse cellular membranes and directly modulate the PD‐1/PD‐L1 signaling pathway.

Furthermore, small molecule inhibitors possess several advantages, including non‐immunogenicity, cost‐effectiveness, oral bioavailability, convenient storage, and transportability. These merits can overcome certain treatment constraints and expand the repertoire of therapeutic possibilities.^[^ ^83^ ^]^ Hence, the exploration and advancement of small‐molecule inhibitors remains promising. Although newly developed small‐molecule compounds exhibit enhanced targeting capabilities, their synthesis process is intricate, and their clinical safety requires further validation. In contrast, repurposing approved drugs can mitigate expenses and substantially shorten the research cycle, given that these drugs have already undergone drug toxicology, pharmacokinetic studies, and clinical trials, thereby accelerating patient benefits.

In this study, we developed a novel network‐based computational framework, **Mnet‐DRI,** to effectively repurpose PD‐L1 modifiers from approved drugs by integrating network proximity, functional similarity, and an RWR‐based network‐diffusion algorithm. Here, we repurposed the five most likely medications, including topiramate, furosemide, oxybutynin, MAP, and clomipramine, to target PD‐L1. Subsequently, we conducted validation experiments on the five identified drugs and observed a significant reduction in the expression of PD‐L1 in colorectal cancer and lung cells upon treatment with MAP. Previous research has indicated that MAP, a tetracyclic antidepressant, exerts its therapeutic effects by blocking the reuptake of norepinephrine by presynaptic membranes to alleviate mental retardation, achieve an antidepressant effect,^[^ ^84^ ^]^ and exhibit visual protection properties.^[^ ^85^ ^]^ Furthermore, many studies have demonstrated that MAP suppresses the development of hepatocellular carcinoma by targeting CRABP1 to inhibit cholesterol biosynthesis.^[^ ^86^ ^]^

Although studies have reported that MAP inhibits tumor growth by downregulating PD‐L1 expression in melanoma,^[^ ^87^ ^]^ the specific mechanisms remain unclear. Furthermore, there are currently no reports on the therapeutic effects of MAP on other cancers. In this study, we found that MAP promotes antitumor effects by targeting SPOP to mediate the proteasomal degradation of PD‐L1. Additionally, MAP demonstrated good efficacy in both colorectal cancer and lung cancer, indicating a certain degree of broad‐spectrum activity. As PD‐L1 expression alone is an unreliable predictive biomarker of the impact of immune checkpoint blockade,^[^ ^88^ ^]^ we also investigated alterations in other immune markers such as CD86, CD80, CD11c, CD206, F4/80, and NK1.1 in mouse tumors. We discovered that the percentage of NK cells, DCs, and M1‐type macrophages within mouse tumor cells increased following MAP treatment, indicating that MAP exerts its anticancer effects by stimulating the immune system. Furthermore, we explored the mechanism by which MAP decreases PD‐L1 expression. Our findings revealed that MAP enhanced PD‐L1 ubiquitination, leading to rapid degradation through interaction with SPOP.

Moreover, our in vitro T‐cell killing assay demonstrated that PD‐L1 knockdown combined with MAP did not produce significant synergistic effects. Given the documented risks associated with anti‐PD‐1 and anti‐CTLA‐4 therapies,^[^ ^89^ ^]^ many investigations have been conducted to explore the synergistic antitumor efficacy of combining small‐molecule drugs with anti‐CTLA4.^[^ ^60^ ^]^ We also conducted animal experiments in which MAP was combined with anti‐CTLA4 therapy. The results revealed that the combined administration of MAP and anti‐CTLA4 exerted a more potent inhibitory effect on tumors than single‐drug administration, solitary use of anti‐CTLA4, or solitary use of anti‐PD‐1. These results demonstrated the feasibility of using MAP as an ICI. Additionally, the limitations of this study should be addressed. First, while we compiled extensive experimentally validated drug‐target interactions and the human interactome, the results may be influenced by potential bias in literature and incomplete data. Second, single‐target drugs might not completely stop all disease‐causing processes, leading to inadequate treatment results.^[^ ^90^ ^]^ Accordingly, it is important to investigate potential alternative targets such as CRABP1 of MAP to exert antitumor effects. Finally, maprotiline can cause adverse effects like sedation, dizziness,^[^ ^91^, ^92^ ^]^ and anticholinergic symptoms such as dry mouth, constipation, and urinary retention in patients with depression and epilepsy.^[^ ^52^, ^93^ ^]^ Therefore, its use necessitates individualized assessments of each patient, particularly concerning their epilepsy and medication history, alongside more frequent and thorough clinical monitoring to ensure safety and effectiveness. Despite these limitations, the **Mnet‐DRI** framework offers substantial benefits for the *in‐silico* drug repurposing of ICP molecules. Moreover, the repurposed drug MAP, a well‐established pharmaceutical agent, possesses evident merits in the adjunctive therapy of colorectal and lung cancers, presenting novel immunotherapeutic alternatives for patients with clinical colorectal and lung cancers.

## 4 Experimental Section

### Construction of the Consolidated Human Interactome

A comprehensive, high‐quality human interactome was constructed by aggregating data from 18 bioinformatics and systems biology databases comprising five experimental assays. These included binary PPIs tested by high‐throughput yeast two‐hybrid (Y2H) systems by integrating two publicly available high‐quality Y2H datasets;^[^ ^94^, ^95^, ^96^ ^]^ kinase‐substrate interactions from the literature‐derived low‐throughput and high‐throughput experiments; PPIs identified through affinity purification followed by mass spectrometry and low‐throughput experiments collated from various databases and literature sources; binary, physical PPIs from protein 3D structures; and signaling networks derived from low‐throughput experiments as annotated in SignaLink2.^[^ ^97^ ^]^ High‐quality PPIs were also constructed from the latest research.^[^ ^98^, ^99^ ^]^ Inferred data, such as gene expression, metabolic associations, and evolutionary analysis data, were excluded. The new version of the consolidated human interactome compiled from these sources contained 18,375 proteins and 485,385 interactions.

### Collection of the Drug‐Target Network and ICI‐Treated Patients with Cancer

Six commonly used databases, namely, Therapeutic Target Database,^[^ ^100^ ^]^ PharmGKB database,^[^ ^101^ ^]^ DrugBank,^[^ ^102^ ^]^ BindingDB,^[^ ^103^ ^]^ ChEMBL,^[^ ^104^ ^]^ and the IUPHAR/BPS Guide to PHARMACOLOGY,^[^ ^105^ ^]^ were collected to collate a reliable set of physical drug‐target interactions. These interactions were defined as those for which binding affinity data, such as the median effective concentration, dissociation constant (Kd), inhibition constant/potency, or median inhibitory concentration (IC_50_), were reported to be 10 µm or less. Eligible interactions were incorporated following three criteria: i) the respective protein targets were required to possess distinct UniProt accession numbers; ii) the protein targets were categorized as “reviewed” in the UniProt database; iii) the protein targets were identified as belonging to the *Homo sapiens* species. After eliminating duplicates, the final network comprised 22498 interactions between 2937 FDA‐approved or investigational drugs and 2883 targets (Table S10, Supporting Information). Moreover, data was collected from four immunotherapy cohorts treated with ICIs in the following studies: i) Hugo et al.,^[^ ^38^ ^]^ ii) VanAllen et al.,^[^ ^39^ ^]^ iii) Riaz et al.,^[^ ^40^ ^]^ and iv) IMvigor210.^[^ ^41^ ^]^ For the Riaz et al. dataset, expression samples collected before drug treatment, during treatment, and from the entire cohort were used. For drug response labels, patients with a partial or complete response were classified as R, while those with stable or progressive disease were classified as NR.

### Identification of PD‐L1‐Associated Genes using the PageRank Algorithm

A PD‐L1‐associated gene module was identified using the PageRank (PR) algorithm. The *PR* value was calculated using the following equation:

(1) $$PR_{i}\left(t\right)=\left(1-d\right)\sum_{j=1}^{n}\frac{a_{ji}}{k_{j}^{out}}PR_{j}\left(t-1\right)+\frac{d}{n}$$

where $a_{ji}/k_{j}^{out}$ denotes the probability that a random walker goes from *v_j_* to *v_i_* in the next step and $k_{j}^{out}$ is the out‐degree of node *v_j_*. In this study, we employed the PageRank algorithm using the NetworkX Python module.^[^ ^106^ ^]^ As an input for the personalization parameter, we assigned a value of one to PD‐L1 and zero to all other genes within the network. Default settings were used for all other parameters. After network propagation, genes with higher association scores were more functionally associated with PD‐L1. The top 200 genes with the highest influence scores were considered as PD‐L1‐associated genes (Table S1, Supporting Information). To evaluate the predictions, several gene sets associated with mechanisms of tumor cell resistance to killing by cytotoxic T cells were collected (Table S11, Supporting Information), including 182 genes from Lawson's studies,^[^ ^107^ ^]^ 74 genes from Li's studies (*P* ≦ 0.05),^[^ ^108^ ^]^ 86 genes from Manguso's studies (*P* ≦ 0.05),^[^ ^109^ ^]^ 325 genes identified with Pmel‐1 T cells and 174 genes identified with OT‐I T cells from Pan's studies (*P* ≦ 0.05).^[^ ^110^ ^]^

### Network Proximity Analysis

To measure the associations between drugs and the PD‐L1‐associated gene module, the “closest” network proximity measure were employed:

(2) $$\langle d_{AB}\rangle =\frac{1}{∥A∥+∥B∥}\;\left(\sum_{a\in A}min_{b\in B^{d}}\left(a,b\right)+\sum_{b\in B}min_{a\in A^{d}}\left(a,b\right)\right)$$

where *d_AB_* is the shortest path between proteins *a* and *b* from protein lists A (drug targets) and B (PD‐L1‐related genes), respectively. To determine the significance of proximity, we normalized the *d* score using a permutation test of 1000 random experiments. For each experiment, the proximity between two randomly generated gene lists with similar degree distributions to A and B were measured. The *Z_NP_* score was calculated as follows:

(3) $$Z_{\mathrm{NP}}=\frac{d-E\left(d_{\mathrm{random}}\right)}{\delta (d_{\mathrm{random}})}$$

where *E*(*d_random_*) and δ(*d_random_*) are the mean and standard deviation of the permutation test, respectively. The significance of the correlation using the 1000 permutations test was evaluated:

(4) $$P=\frac{\left\{d_{\mathrm{random}}<d\right\}}{1000}$$

A nominal *P‐*value was calculated for each predicted compound by computing the number of observed *d* values greater than the permutations *d_random_*. Drugs with *Z_NP_* < −3 and *P* < 0.05 were considered significantly proximal.

### FS Analysis

FS analyses were conducted using Wang's method combined with the best‐matched average strategy in the R package GoSemSim.^[^ ^111^ ^]^ The FS score between PD‐L1‐associated genes and drug targets were calculated in the BP, MF, and CC aspects of the GO terms to achieve a comprehensive characterization of gene function. The FS score was calculated as follows:

(5) $$FS=\frac{\left(Sim_{BP}+Sim_{MF}+Sim_{CC}\right)}{3}$$

Furthermore, 1000 random experiments were performed to evaluate the significance of functional similarity.

(6) $$P=\frac{\left\{FS_{\mathrm{random}}>\mathrm{FS}\right\}}{1000}$$

Drugs with *FS* > 0.6 and *P* < 0.05 were considered to have significant functional similarities.

### RWR‐Based Network Diffusion Analysis

The RWR‐based network diffusion algorithm was used to evaluate the efficacy of a drug for a specific disease.^[^ ^112^, ^113^ ^]^ Inspired by this idea, this method was used to screen drugs that inhibit PD‐L1 expression. First, drug targets and PD‐L1‐associated genes were used as seed nodes to run the RWR algorithm in the PPI network. A random walk with a restart is defined as follows:

(7) $$P_{t+1}=\left(1-\gamma \right)Wp_{t}+\gamma p_{0}$$

where *p*_0_ is the initial probability vector in which equal probabilities are assigned to the starting nodes (PD‐L1 or drug targets); *p_t_* represents the probability vector comprising probabilities assigned to nodes at step *t*; *γ* signifies the restarting probability; *W* represents the transition matrix, which is essentially a column‐normalized adjacency matrix of the network. Beginning with the initial set of nodes within the network, the walker iteratively advances from the current nodes to randomly selected neighboring nodes or returns to the initial nodes. When stability is reached through iterations (when the change between *p_t_* and *p*_*t* + 1_ falls below 10^−30^), the probability vector can be utilized to express the association scores of all genes in the network concerning the starting genes.

Using this methodology, the influence score vectors for both sets of seed nodes for all nodes within the PPI network was derived. Subsequently, the Pearson correlation coefficient (*Cor*) was computed between the two score vectors. To ascertain the statistical significance of this correlation, a permutation test with 1000 iterations was conducted. Then the following formula was utilized to calculate *Z_RWR_* and its associated *P* value:

(8) $$Z_{\mathrm{RWR}}=\frac{\mathrm{Cor}-E\left(\mathrm{Co}r_{\mathrm{random}}\right)}{\delta \left(\mathrm{Co}r_{\mathrm{random}}\right)}$$

(9) $$P=\frac{\left\{Cor_{random}>Cor\right\}}{1000}$$

where *E*(*Cor_random_*) and δ(*Cor_random_*) are the mean and standard deviation of the Pearson correlation coefficients between the influence score vectors of the drug targets, respectively. The *P‐value* was calculated according to the permutation test. The RWR algorithm was executed using the *dnet* package, and the default parameters were used for subsequent analysis.^[^ ^114^ ^]^ Drugs with a Z_RWR_ > 3 and *P* < 0.05 were considered candidates.

### Tissue‐Specific Analysis and Evaluation of Immunological Characteristics

The specificity of gene expression was assessed in colorectal tissue in 32 different tissues using RNA‐Seq data sourced from the GTEx database.^[^ ^115^ ^]^ Details of data processing are provided in previous studies.^[^ ^116^ ^]^ The expression specificity of gene *i* in tissue *t* was defined as follows:

(10) $$Z_{\left(i,t\right)}=\frac{E\left(i,t\right)-E\left(i\right)}{\delta_{i}}$$

Where *E*(*i*) is the mean and δ_*i*_ is the standard deviation of the expression of gene *i* across all tissues, and *E*(*i*, *t*) is the mean expression of gene *i* in tissue *t*. Furthermore, gene expression data of TCGA‐COAD, TCGA‐READ, and TCGA‐LUAD samples were downloaded from https://toil‐xena‐hub.s3.us‐east‐1.amazonaws.com/download/tcga_RSEM_gene_tpm.gz. The values were standardized by transcripts per kilobase million (TPM) and expressed as log2(TPM + 0.001). Moreover, the immunological characteristics of the tumor microenvironment, including the activity of the cancer immune cycle, infiltration level of tumor‐infiltrating immune cells, effector genes of tumor‐infiltrating immune cells, and expression of immunomodulators, were analyzed. The cancer immune cycle reflects the anticancer immune response and comprised seven steps. The activities were qualified using single‐sample gene set enrichment analysis.^[^ ^117^ ^]^ The levels of tumor‐infiltrating immune cells were calculated using the TIMER algorithm. In addition, information on 31 effector genes of tumor‐infiltrating immune cells and 114 immunomodulators were collected from previous studies.^[^ ^118^ ^]^

### Reagents and Cell Culture

MAP hydrochloride, primaquine, MG‐132, Baf, CQ, CHX, and 3‐MA were obtained from MedChemExpress (Monmouth Junction, NJ, USA). The antibodies used are listed in Table S12 (Supporting Information). RKO, HT29, DLD1 human colorectal cancer cells, MC38 mouse colorectal cancer cells, and LLC Lewis and H1975 cells were obtained from the Shanghai Institute of Cell Biology, Chinese Academy of Sciences (Shanghai, China). PD‐1‐overexpressing Jurkat cells were donated by the Kongming Wu Research Group (Department of Oncology, Tongji Hospital of Tongji Medical College, Huazhong University of Science and Technology, Wuhan, China). RKO cells were cultured in MEM. MC38 and H1975 cells were cultured in DMEM. HT29 cells were cultured in McCoy's 5A medium. DLD1, Lewis, and Jurkat cells were cultured in the RPMI 1640 medium. All culture media were acquired from Meilunbio (Dalian, China). Subsequently, the culture media was supplemented with 100 mg mL^−1^ streptomycin, 100 U mL^−1^ penicillin, and 10% fetal bovine serum (Biological Industries, Cromwell, CT, USA), and the cells were incubated in a carbon dioxide (CO_2_) incubator containing 5% CO_2_.

### Cell Viability and Toxicity Assays

Cell viability was determined using the CCK‐8 assay. First, the cells were seeded in a flat‐bottom 96‐well plate at 8 × 10^3^ cells/well density. Subsequently, the cells were cultured until they had completely adhered. Afterward, the cells were treated with the designated concentrations of drugs, and the drug treatment was continued for 24 h. After treatment, 10 µL of CCK‐8 reagent from Beyotime (Haimen, China) was added to each well, and the plate was incubated at 37°C for ≈3 h. Finally, the absorbance at 450 nm was measured using an enzyme‐linked immunosorbent assay reader, and the IC_50_ was calculated using the logit method.

An appropriate number of cells was first cultured in a 12‐well plate to assess the impact on cell proliferation. Once the cells had fully adhered, they were treated with MAP for 24 h. Subsequently, the cells were incubated with the EdU working solution for 2 h, followed by fixation, washing, permeabilization, and staining. Finally, a high‐content cell‐imaging analysis system was used for detection. The cell proliferation assay kit used in the experiment was bought from Biyotime (Haimen, China). For the specific steps, refer to the instructions provided by the manufacturer.

### Western Blotting and Immunoprecipitation

The cells were inoculated at a density of 4 × 10^5^ cells/well in a 6‐well plate. After 24 h, the cells were treated with the drugs, and all cells were collected after an appropriate period of drug exposure. Total protein was extracted using RIPA lysis buffer supplemented with 1% protease inhibitor (Beyotime, Haimen, China). The protein concentration was measured using a BCA Protein Assay Kit (Beyotime, Haimen, China). Proteins were separated by SDS‐PAGE and transferred onto a PVDF membrane. After blocking with 5% skim milk, the membrane was incubated with specific antibodies overnight at 4°C, followed by incubation with secondary antibodies at room temperature for 1 h. The cells were scanned using a Bio‐Rad imaging system. For immunoprecipitation experiments, collected cells were lysed with IP cell lysis buffer containing 1% protease inhibitor (Beyotime, Haimen, China). The lysate was incubated with anti‐FLAG beads overnight at 4°C on a shaker, and protein blot analysis was performed after washing five times. The pertinent antibodies used are listed in Table S7 (Supporting Information), and the dilution instructions followed the manufacturer's instructions.

### Flow Cytometry and Immunofluorescence

The cells were treated with MAP for a specified time, collected, and incubated with an anti‐PD‐L1 antibody at 4 °C for 30 min. Afterward, the cells were washed with phosphate‐buffered saline (PBS), and the washed live cells were resuspended in 500 µL of PBS. The abundance of PD‐L1 on the cell membrane was detected using flow cytometry. Besides, PD‐L1 expression in the cell membrane was detected using immunofluorescence. The cells were seeded in a 12‐well plate and treated with MAP after complete adhesion. After a specific duration of treatment, fixation, blocking, and antibody incubation were performed, and images were captured using a Cytation 5‐cell imaging microplate detection system. The antibodies used are listed in Table S12 (Supporting Information).

### RT‐PCR Analysis

RNAiso‐Plus (Takara, Dalian, China) was used to extract total RNA from cells. Total mRNA was reverse‐transcribed into cDNA using the Prime Script RT Kit (Takara, Shiga, Japan) following the manufacturer's instructions. RT‐PCR was performed using a LightCycler 96 (Roche, Basel, Switzerland) with β‐actin as the reference gene. The primer sequences are listed in Table S13 (Supporting Information).

### Transfection

The siRNAs for gene knockdown were acquired from GenePharma (Shanghai, China). A negative control (NC) was used as a control. siRNA double strands were transfected using Lipofectamine 2000 (Invitrogen, Carlsbad, CA). The cell culture medium was replaced 8 h after transfection, and the cells were cultured for 48 h before drug treatment for 24 h. The relevant siRNA sequences are listed in Table S14 (Supporting Information). Transfected plasmid pcDNA3.1‐Ub was procured from GenePharma (Shanghai, China). The other steps and reagents used were the same as those for the siRNA transfection.

### T‐Cell‐Mediated Tumor Cell Killing Assay

RKO and H1975 cells were seeded in a 12‐well plate at 2.5 × 10^5^ cells/well density. The cells were cultured until fully adhered, and MAP was administered for 24 h. Subsequently, Jurkat cells stably transfected with human PD‐1 (activated with 1 mg mL^−1^ phytohemagglutinin plus 50 mg mL^−1^ phorbol 12‐myristate 13‐acetate) were introduced at a ratio of 1:9. After 48 h, crystal violet staining was used to identify the surviving tumor cells, and images were captured using Cytation 5 (BioTek, USA).

### CETSA

RKO cells were treated with RIPA buffer to break them down. The resulting protein extracts were divided into two parts. One part was treated with 200 µm MAP, while the other received an equal amount of DMSO. Both the mixtures were incubated at room temperature for 25 min. The samples were then divided into 100 µL PCR tubes and exposed to a range of temperatures, starting at 25 °C. After cooling and spinning, 5× SDS loading buffer was added to the liquid above the solid residue. The samples were then heated to 95 °C for 10 min and tested using Western blotting using SDS‐PAGE gels.

### MST

GFP target protein plasmids were constructed, and GFP SPOP or mutant plasmids were used to overexpress the proteins in 293T cells. After 48 h, the cells were lysed with IP lysis buffer to obtain cell lysates. Assays were performed using a MonolithTM NT.115 An MST device (NanoTemper, Germany) was used.

### Animal Experiments

All animal experiments were conducted following the ethical obligations of the Department of Laboratory Animal Science, Shanghai University of Traditional Chinese Medicine (SHUTCM). Approval No: PZSHUTCM2303030004; PZSHUTCM2305310005. Female C57BL/6J mice and nude mice aged 6–8 weeks were obtained from the Shanghai Jihui Laboratory Animal Breeding Co. (Shanghai, China). MC38 (8 × 10^5^ cells) and Lewis (3 × 10^7^ cells) were inoculated subcutaneously into C57BL/6J mice and the axillary region of the nude mice, respectively. When the tumor volume reached 50 mm^3^, the mice in the antibody treatment groups were administered intraperitoneal injections of anti‐PD‐1 (100 µg) or anti‐CTLA4 (100 µg) every 5 days for 3 doses. For the MAP treatment groups, gavage was used for drug administration. Tumor measurements were taken every other day using a caliper, and the tumor volume was calculated using the following formula: ½ × length × width2. After the experiment, a portion of the tumor tissue from each group was collected for immunohistochemical and flow cytometry analysis, and the major organs were collected for HE staining. Detailed information regarding the relevant antibodies is provided in Table S11 (Supporting Information).

### Tumor‐Infiltrating Lymphocyte Isolation and T‐Cell Profiling

Tumor tissues from different groups of mice were collected, cut into small pieces, and digested using collagenase type 4 (1 mg mL^−1^, Yeasen) and DNAzyme 1 (0.1 mg mL^−1^, Yeasen) for 2 h at 37 °C. The cells were then incubated with surface‐labeled antibodies against CD3, CD8, CD25, Gr‐1, CD11b, GzmB, and Foxp3 for 30 min at 4 °C. After staining, the cells were washed with PBS containing 2% serum, and all samples were analyzed using flow cytometry (Beckman–Coulter, USA). Data analysis was performed using FlowJo software, and the relevant antibodies used are listed in Table S12 (Supporting Information).

### Statistical Analysis

All analyses were performed using the R software (version 4.3.1; http://www.r‐project.org/) and Python software (version 3.9.13; https://www.python.org/). The data from the biological verification section were expressed as the mean ± standard error of the mean and were statistically analyzed and graphed using GraphPad Prism software (version 9.0.1), with an independent samples t‐test for two‐by‐two comparisons and one‐way analysis of variance (ANOVA) for comparisons of multiple datasets. Tumor growth data from the mice were analyzed using two‐way ANOVA with repeated measures. Quantification of the data was performed using ImageJ‐win64. Human colon cancer and adjacent tissues were acquired from the Century Forum Hospital of Capital Medical University. However, human lung cancer and adjacent tissues were obtained from the Longhua Hospital of Shanghai University of Traditional Chinese Medicine.

### Code Availability

The main code for Mnet‐DRI analysis is available at https://github.com/saisaitian/Mnet‐DRI. The other codes written for and used in this study are available from the corresponding author upon reasonable request.

## Conflict of Interest

The authors declare no conflict of interest.

## Author Contributions

S.T., M.X., and X.G. contributed equally to this work. W.Z., S.L., and Q.W. performed conceptualization, original draft, methodology, did editing and reviewed the manuscript, funding acquisition, and supervision. S.T., M.X., X.G., and J.F. analyzed the data, carried out the experiments, generated the figures, and wrote the paper. H.X., X.X., H.H., Q.Z., D.Y., M.C., Y.Z., J.X., M.G., H.Z., J.L., and Y.G. participated in part of the experiments.

## Supporting information



Supporting Information

Supplemental Table 1

Supplemental Table 7

## Data Availability

The data that support the findings of this study are available from the corresponding author upon reasonable request.
