# Supplementary material for: Network Medicine‐Based Strategy Identifies Maprotiline as a Repurposable Drug by Inhibiting PD‐L1 Expression via Targeting SPOP in Cancer
Source: Adv Sci (Weinh). 2024 Nov 5;12(1):2410285. doi: 10.1002/advs.202410285 (PMC11714211; doi:10.1002/advs.202410285)
Supplement: Supplementary file 1 — Supporting Information [file ADVS-12-2410285-s001.docx]

**Supplementary Information**

**Network medicine-based strategy** **identifies** **maprotiline as a repurposable drug by inhibiting PD-L1 expression via targeting SPOP in cancer**

Saisai Tian^1†^, Mengting Xu^2†^, Xiangxin Geng^2†^, Jiansong Fang^3^, Hanchen Xu^4^, Xinying Xue^5^, Hongmei Hu^2^, Qing Zhang^2^, Dianping Yu^2^, Mengmeng Guo^2^, Hongwei Zhang^2^, Jinyuan Lu^1^, Yangcheng Guo^1^, Qun Wang^2*^, Sanhong Liu^2*^, Weidong Zhang^1,6,7*^

^1^ Department of Phytochemistry, School of Pharmacy, Second Military Medical University, Shanghai, China

^2^ Shanghai Frontiers Science Center of TCM Chemical Biology, Institute of Interdisciplinary Integrative Medicine Research, Shanghai University of Traditional Chinese Medicine, Shanghai, China

^3^ Science and Technology Innovation Center, Guangzhou University of Chinese Medicine, Guangzhou, China

^4^ Institute of Digestive Diseases, Longhua Hospital, Shanghai University of Traditional Chinese Medicine, Shanghai, China

^5^ Department of Respiratory and Critical Care, Emergency and Critical Care Medical Center, Beijing Shijitan Hospital, Capital Medical University, Beijing, China

^6^ State Key Laboratory for Quality Ensurance and Sustainable Use of Dao-di Herbs, Institute of Medicinal Plant Development, Chinese Academy of Medical Sciences and Peking Union Medical College, Beijing, China

^7^ The Research Center for Traditional Chinese Medicine, Shanghai Institute of Infectious Diseases and Biosafety, Institute of Interdisciplinary Integrative Medicine Research, Shanghai University of Traditional Chinese Medicine, Shanghai, China

† These authors contributed equally to this work.

***Corresponding authors:**

**Weidong Zhang, Ph.D., Professor**

Address: School of Pharmacy, Second Military Medical University, 325 Guohe Road, Shanghai, 200433, China

E-mail: wdzhangy@hotmail.com

**Sanhong Liu, Ph.D., Professor**

Address: Institute of Interdisciplinary Integrative Medicine Research, Shanghai University of Traditional Chinese Medicine, 1200 Cailun Road, Shanghai, 201203, China

E-mail: liush@shutcm.edu.cn

**Qun Wang, Ph.D., Associate Professor**

Address: Institute of Interdisciplinary Integrative Medicine Research, Shanghai University of Traditional Chinese Medicine, 1200 Cailun Road, Shanghai, 201203, China

E-mail: qunwang0523@163.com


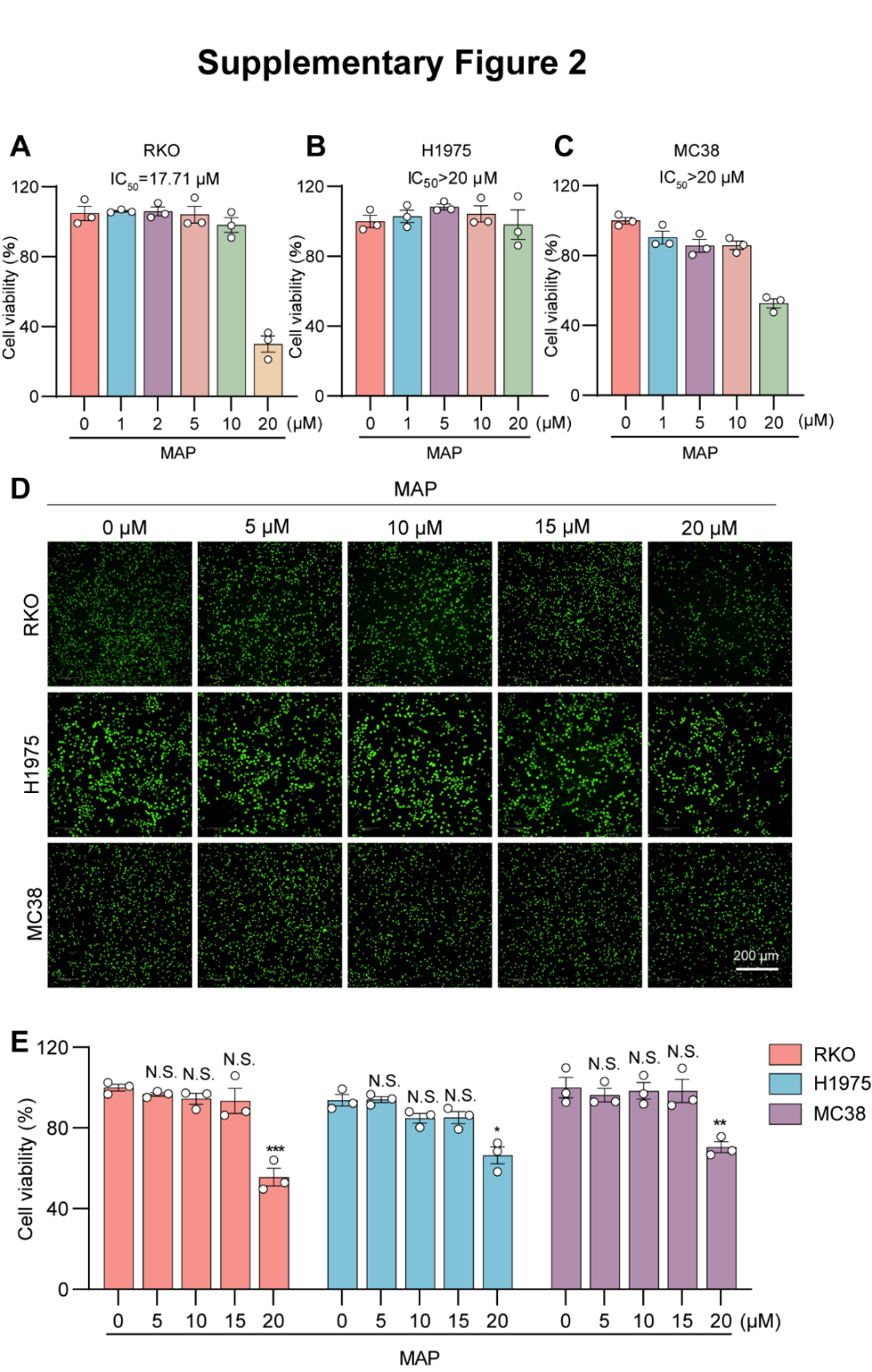


**Figure S1. MAP is not cytotoxic at effective concentrations. (A-C)** The inhibitory effects of MAP on RKO (A), H1975 (B) and MC38 (C) cells were detected with a CCK-8 kit. **(D)** The toxicity of MAP at different concentrations on RKO, H1975 and MC38 colorectal cancer cells was detected via an EdU kit. **(E)** Quantification of the green fluorescence intensity in (D). The data shown are the mean ± standard error of the mean (SEM). Statistical differences were determined by Student’s t test. **p < 0.01; ***p < 0.001; N.S., not significant.

**
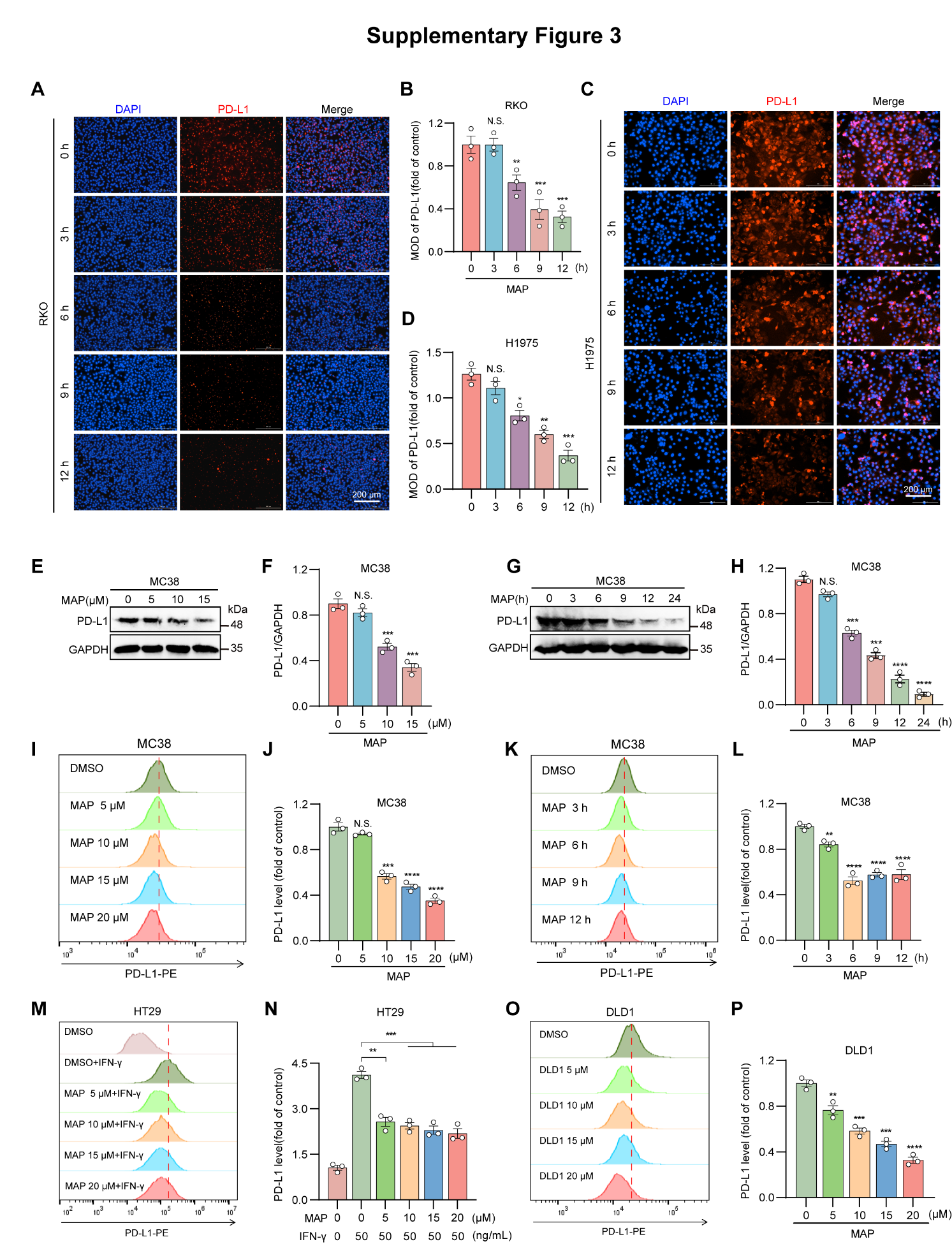
**

**Figure S2.** **MAP suppresses PD-L1 expression in cancer cells. (A-D)** RKO (A) and H1975 (B) cells were stained with DAPI and treated with MAP (10 μM) for different time periods. The scale bar is 200 μm. (B) and (D) Quantitative analysis of the data in (A) and (C), respectively. **(E-H)** Western blotting was used to detect PD-L1 levels in MC38 cells after treatment with different concentrations of MAP for 24 h (E) or treatment with 10 μM MAP for different durations (G). (F) and (H) Quantitative results of (E) and (G), respectively. **(I-L)** Detection of membrane PD-L1 levels in MC38 cells after treatment with different concentrations of MAP for 24 h (I) or treatment with 10 μM MAP for different durations (K) by flow cytometry. (J) and (L) Quantitative results of (I) and (K), respectively. **(M-P)** Flow cytometry was used to detect the inhibitory effect of different concentrations of MAP on the membrane expression of PD-L1 in HT29 (M) and DLD1 (O) colorectal cancer cells. (N) and (P) represent the quantitative results of (M) and (O), respectively. The data shown are the mean ± standard error of the mean (SEM). Statistical differences were determined by Student’s t test. *p < 0.05; **p < 0.01; ***p < 0.001; ****p < 0.0001; N.S., not significant.


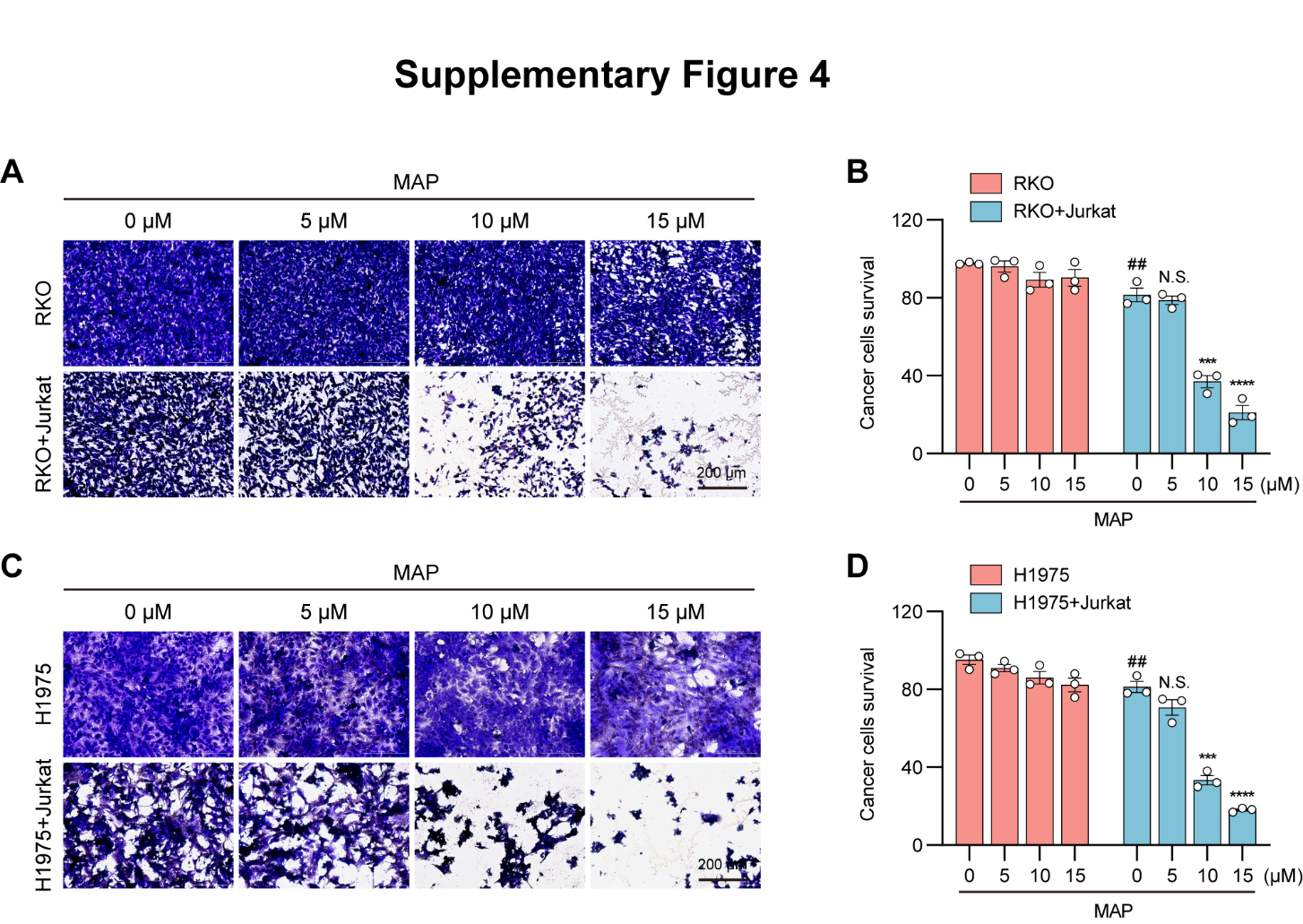


**Figure S3. MAP can enhance the killing ability of T cells *in vitro*. (A)** Jurkat cells were cocultured with RKO and H1975 cells treated with MAP at different concentrations in a 12-well plate for 48 hours, and the surviving cells were observed through crystal violet staining. **(B)** Relative fold increase in surviving cells. The data shown are the mean ± standard error of the mean (SEM). Statistical differences were determined by Student’s t test. ***p < 0.001; ****p < 0.0001; N.S., not significant.

**
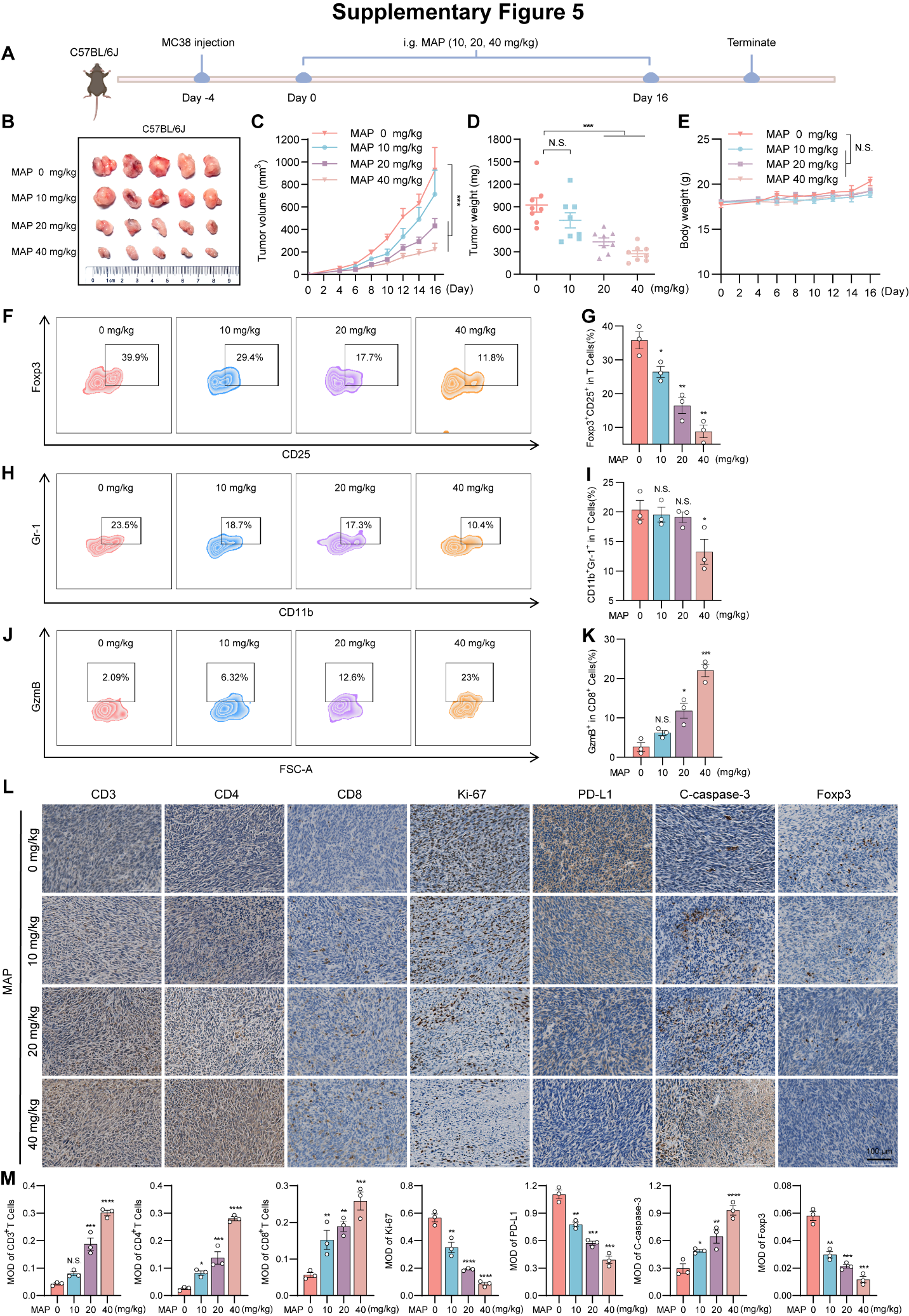
**

**Figure S4. MAP inhibits tumor growth by enhancing the cytotoxicity of T cells. (A-E)** C57BL/6J (female) mice were treated with corn oil and MAP (10, 20, and 40 mg/kg) *via* oral gavage after subcutaneous inoculation of MC38 colorectal cancer cells (8×10^5^ cells/mouse); n = 8 mice per group. (A) Scheme representing the intragastric experimental procedure. (B) Representative solid tumors excised from mice in different groups. (C) Tumor growth curves of mice in different groups. (D) Tumor weights excised from mice in different groups. (E) Changes in the body weights of the mice in each group were recorded. **(F-K)** Representative flow cytometry plots of CD4^+^CD25^+^Foxp3^+^ (F), CD11b^+^Gr-1^+^ (H), and GzmB expression in CD3^+^CD8^+^TILs (J) from MC38 tumors treated with different therapeutic regimens. The quantitative results are shown in panels (G), (I), and (K). **(L and M)** Immunohistochemical staining showing the expression of CD3, CD4, CD8, Ki-67, PD-L1, C-caspase-3, and Foxp3 in MC38 tumor tissues from mice in different groups. The scale bar represents 100 μm in panel (L). The quantitative results for panel (L) are shown in panel (M). The data shown are the mean ± standard error of the mean (SEM). Tumor growth data from the mice were analyzed by two-way ANOVA with repeated measures. Statistical differences in other data were determined by Student’s t test. *p < 0.05; **p < 0.01; ***p < 0.001; ****p < 0.0001; N.S., not significant.


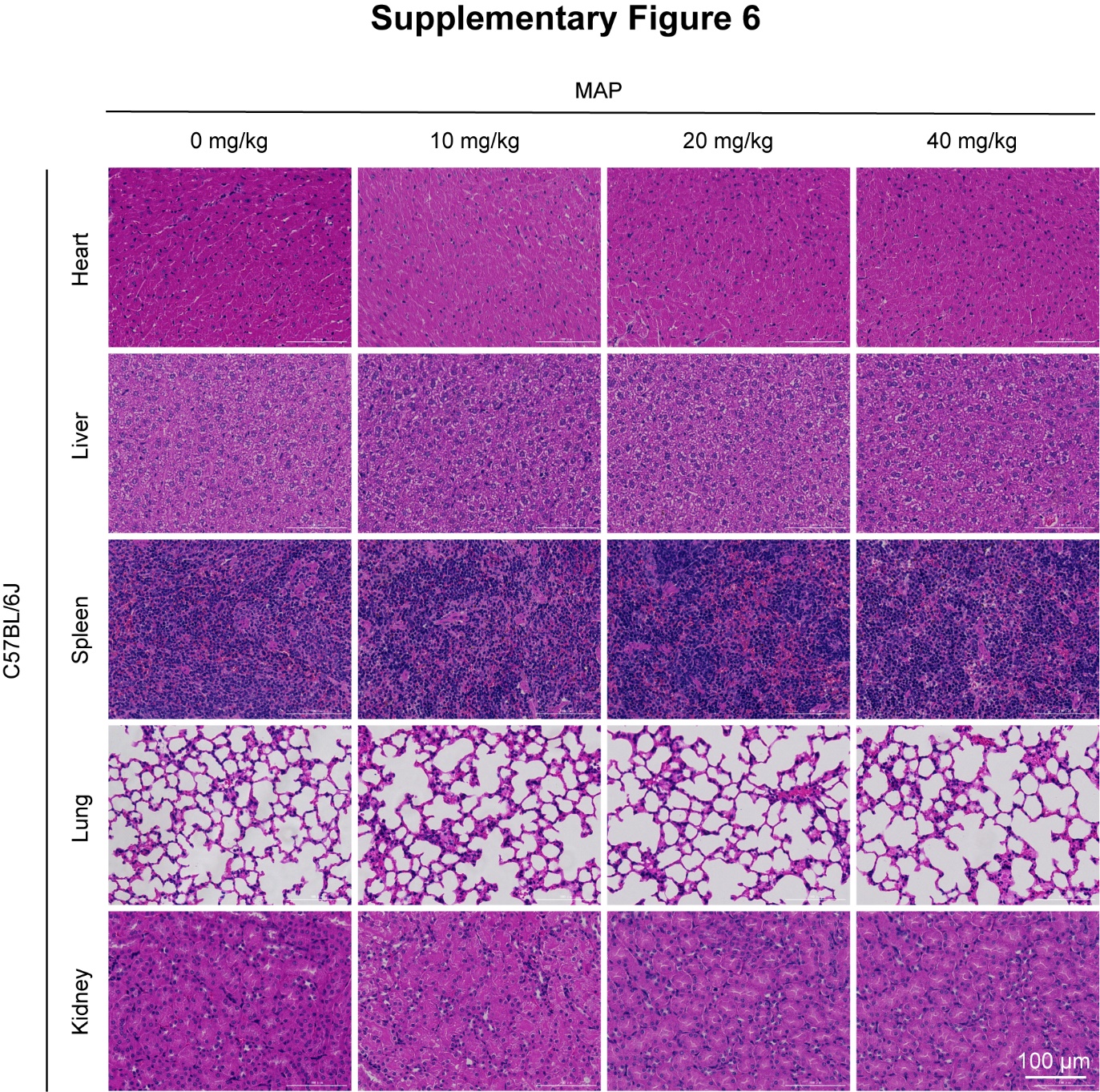


**Figure S5.** **MAP has no toxic side effects on mice.** MC38 colorectal cancer cells were subcutaneously inoculated into C57BL/6J (female) mice with corn oil and MAP (10, 20, or 40 mg/kg) *via* oral gavage (n=8). H&E staining was conducted on heart, liver, spleen, lungs, and kidneys extracted from mice in different groups.


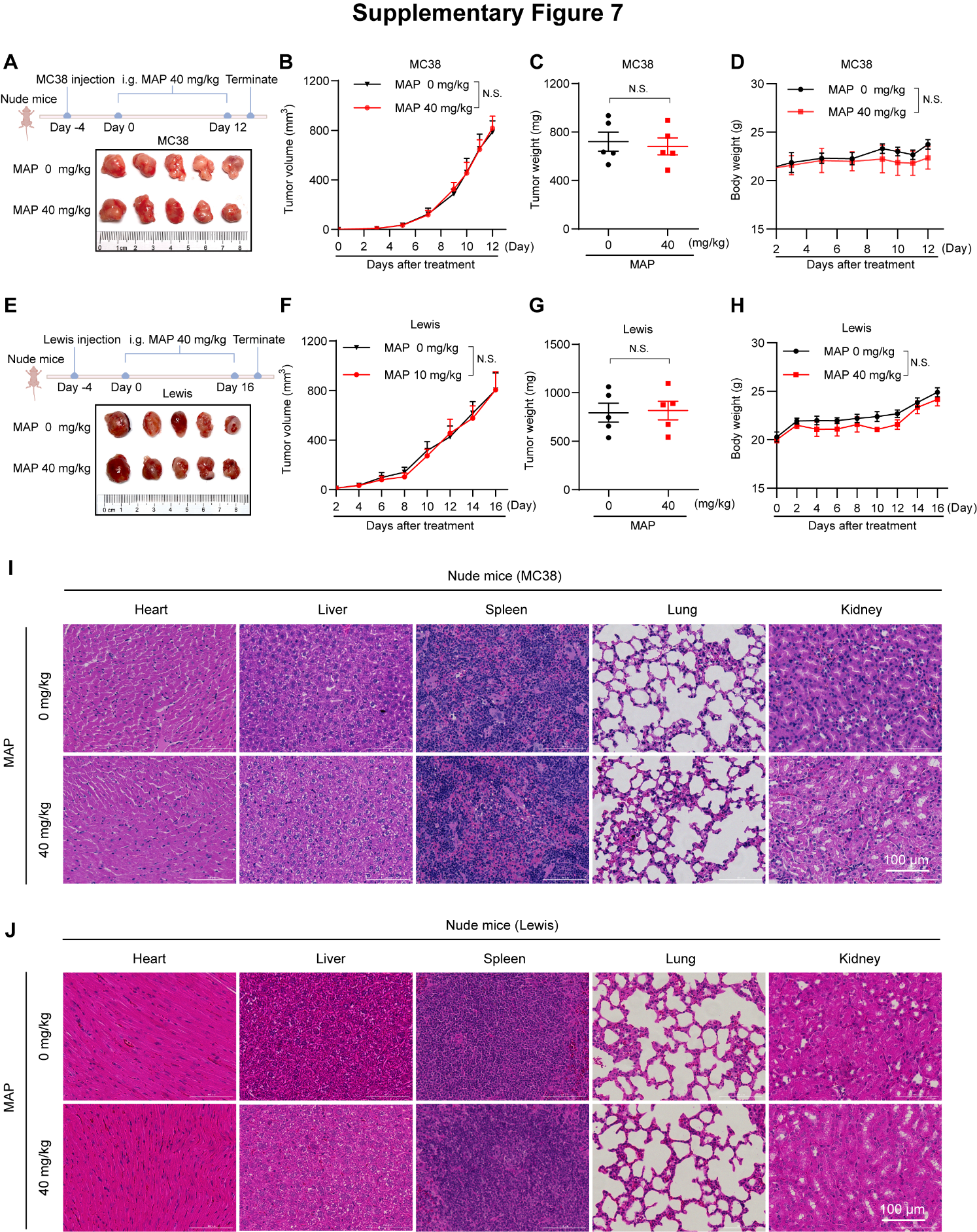


**Figure** **S6. MAP has no antitumor effect on nude mice. (A-H)** Colorectal cancer MC38 cells or Lewis lung cancer cells were inoculated into the armpits of female nude mice (8×10^5^ MC38 cells or 3×10^6^ Lewis cells per mouse) to establish a subcutaneous tumor model. The mice were orally administered corn oil or MAP (40 mg/kg) for treatment; n = 5 mice per group. (A and E) Schematic diagrams of the experimental procedure (i.e., intragastric experiments) and representative solid tumors from nude mice. (A) Diagram of tumors from nude mice inoculated with MC38 cells. (E) Diagram of tumors from nude mice inoculated with Lewis cells. (B and F) Tumor growth curves of various treatment groups of nude mice injected with MC38 or Lewis cells (B) and (F). (C and G) Tumur weights of various treatment groups of nude mice injected with MC38 cells or Lewis cells, denoted as (C) and (G), respectively. (D and H) Changes in body weight during drug administration in nude mice injected with MC38 (D) or Lewis (H) cells. **(I-J)** HE staining of heart, liver, spleen, lung, and kidney tissues from nude mice injected with MC38 cells (I) or Lewis cells (J) and from nude mice in the corn oil and MAP (40 mg/kg) groups. The data shown are the mean ± standard error of the mean (SEM). Tumor growth data from the mice were analyzed by two-way ANOVA with repeated measures. Statistical differences in other data were determined by Student’s t test. N.S., not significant.


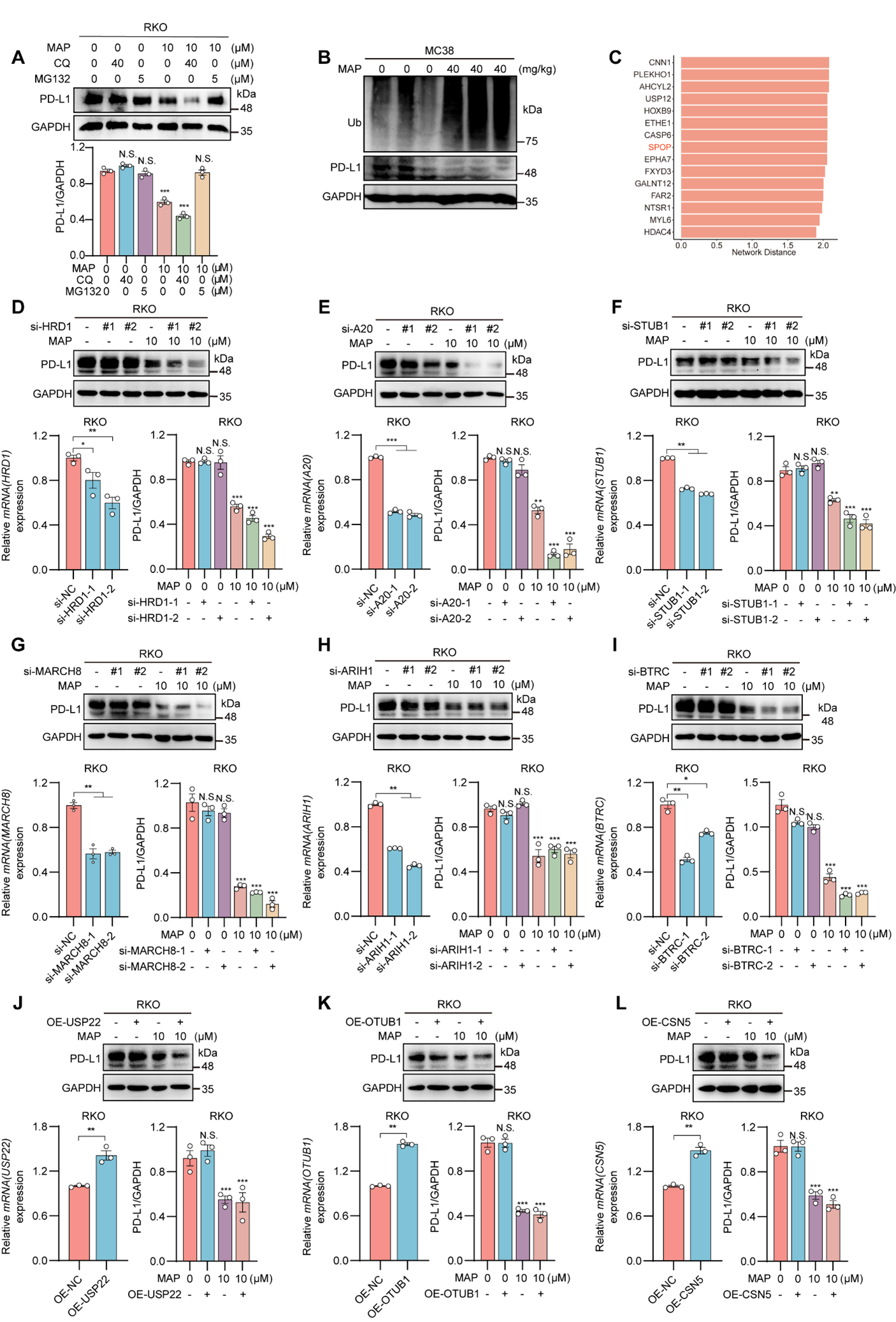


**Figure** **S7.** **E3 Ubiquitin ligase enzymes or DUBs** **in addition to SPOP do not reverse the decrease in PD-L1 caused by MAP. (A)** RKO cells were cotreated with lysosome inhibitor CQ (40 μM), proteasome inhibitor MG132 (5 μM), or MAP (10 μM) for 12 h, after which PD-L1 expression was detected via Western blotting. **(B)** Western blotting detected ubiquitination levels and PD-L1 levels in tumor tissues of MC38 subcutaneous tumor mice treated with corn oil or MAP (40 mg/kg). **(C)** The network distance (the shortest path) of top 15 proximity genes (colon-specific expression) to the PD-L1 gene module in the human protein–protein interactome. **(D-I)** Western blotting was performed to investigate whether PD-L1 remained downregulated in RKO cells following MAP (10 μM) treatment via siRNA interference with HRD1 (C), A20 (D), STUB1 (E), MARCH8 (F), ARIH1 (G), or BTRC (H). The knockdown efficiency was then determined by RT-qPCR. **(J-L)** Western blotting was performed to determine whether PD-L1 was downregulated in RKO cells after MAP (10 μM) treatment with USP22 (I)、OTUB1 (J) or CSN5 (K) overexpression, and the knockdown efficiency was evaluated using RT-qPCR. The data shown are the mean ± standard error of the mean (SEM). Statistical differences were determined by Student’s t test. *p < 0.05; **p < 0.01; ***p < 0.001; N.S., not significant.


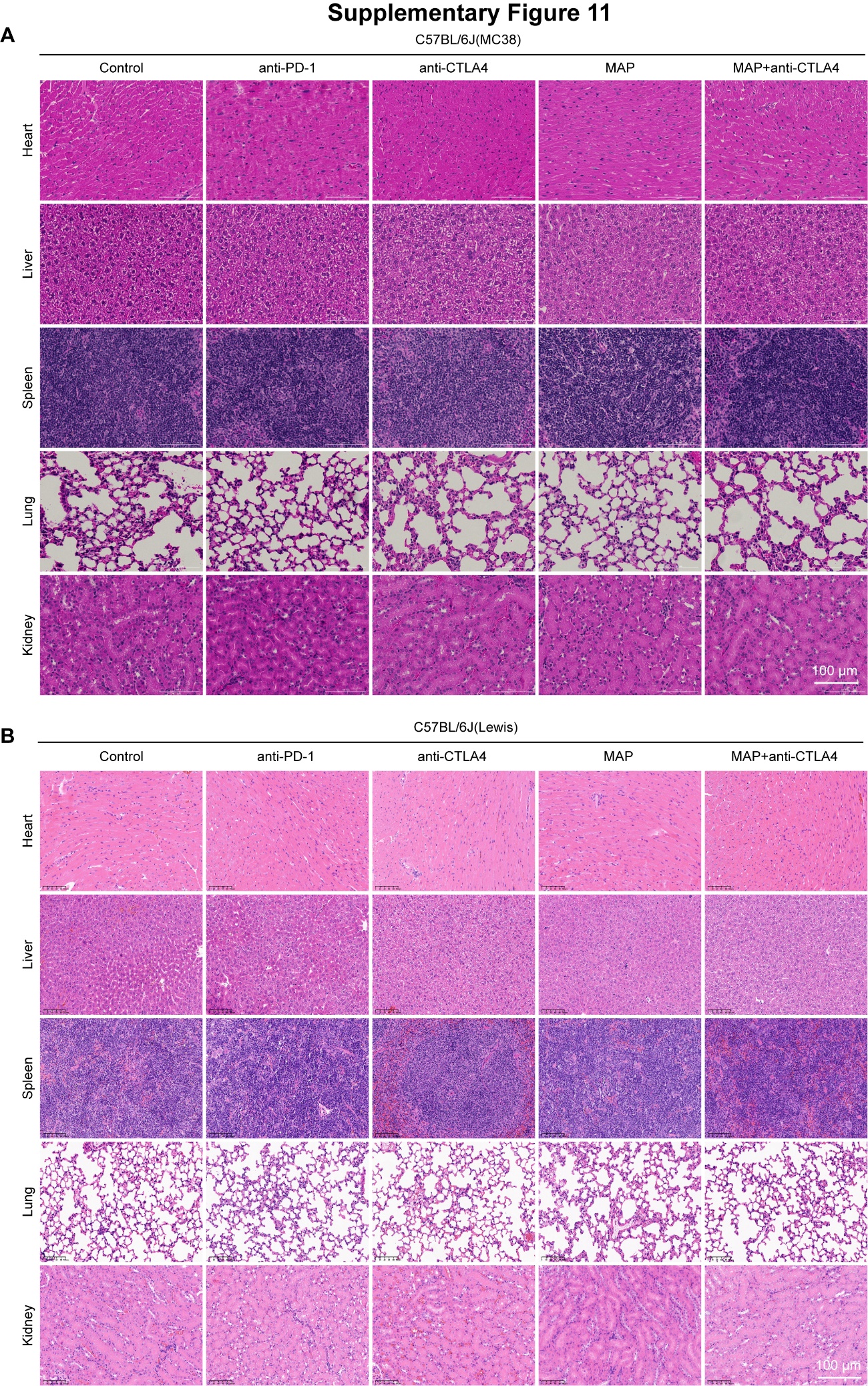


**Figure S8. MAP and anti-CTLA4 antibodies were** **combined to treat colon or lung cancer mice without toxic side effects. (A-B)** C57BL/6J (female) mice were orally treated with corn oil, anti-PD-1, anti-CTLA4, MAP (40 mg/kg), or MAP (40 mg/kg) in combination with anti-CTLA4 after subcutaneous inoculation of MC38 colorectal cancer cells (8×10^5^ cells/mouse) or Lewis lung cancer cells (3×10^6^ cells/mouse), n = 5 mice per group. H&E staining of the heart, liver, spleen, lungs and kidneys of different groups of mice with colon (A) or lung (B) cancer.


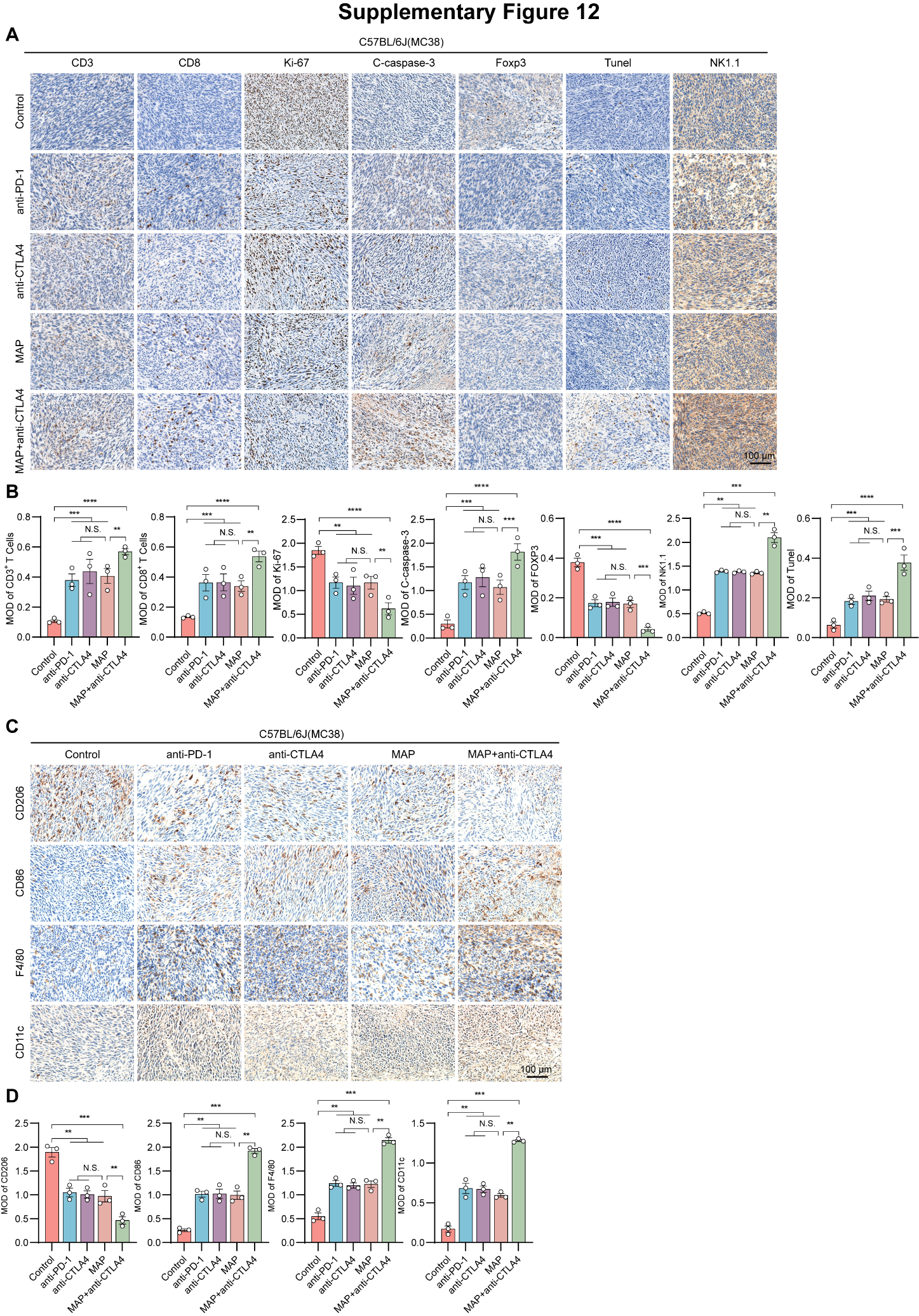


**Figure S9. MAP enhances immune cell infiltration in the tumor tissue of mice injected with colon cancer cells. (A-D)** C57BL/6J (female) mice were orally treated with corn oil, anti-PD-1, anti-CTLA4, MAP (40 mg/kg), or MAP (40 mg/kg) in combination with anti-CTLA4 after subcutaneous inoculation of MC38 colorectal cancer cells (8×10^5^ cells/mouse), n = 5 mice per group. Immunohistochemical staining was used to analyze the expression of CD3, CD8, Ki-67, C-caspase-3, Foxp3, TUNEL and NK1.1 in the tumor tissues of mice in each group (A), and (B) is a quantification of (A). CD206, CD86, F4/80 and CD11c (C) in tumor tissue were also analyzed by immunohistochemical staining and quantified (D). The data shown are the mean ± standard error of the mean (SEM). Statistical differences were determined by Student’s t test. *p < 0.05; **p < 0.01; ***p < 0.001; ****p < 0.0001; N.S., not significant.


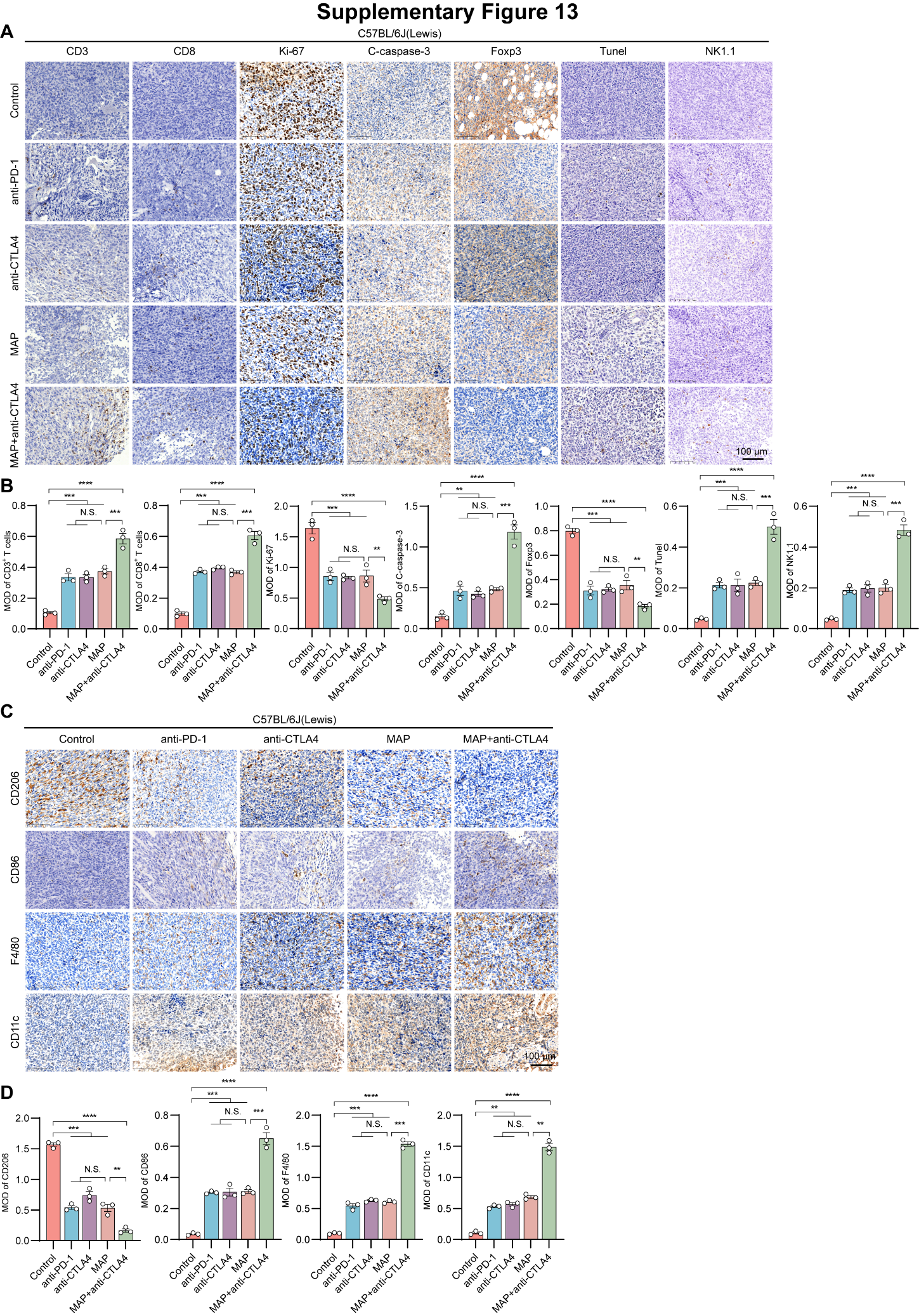


**Figure S10. MAP enhances immune cell infiltration in the tumor tissue of mice injected with lung cancer cells (A-D)** C57BL/6J (female) mice were orally treated with corn oil, anti-PD-1, anti-CTLA4, MAP (40 mg/kg), or MAP (40 mg/kg) in combination with anti-CTLA4 after subcutaneous inoculation of Lewis lung cancer cells (3×10^6^ cells/mouse), n = 5 mice per group. Immunohistochemical staining was used to analyze the expression of CD3, CD8, Ki-67, C-caspase-3, Foxp3, TUNEL and NK1.1 in the tumor tissues of mice in each group (A), and (B) is a quantification of (A). CD206, CD86, F4/80 and CD11c (C) in tumor tissue were also analyzed by immunohistochemical staining and quantified (D). The data shown are the mean ± standard error of the mean (SEM). Statistical differences were determined by Student’s t test. *p < 0.05; **p < 0.01; ***p < 0.001; ****p < 0.0001; N.S., not significant.


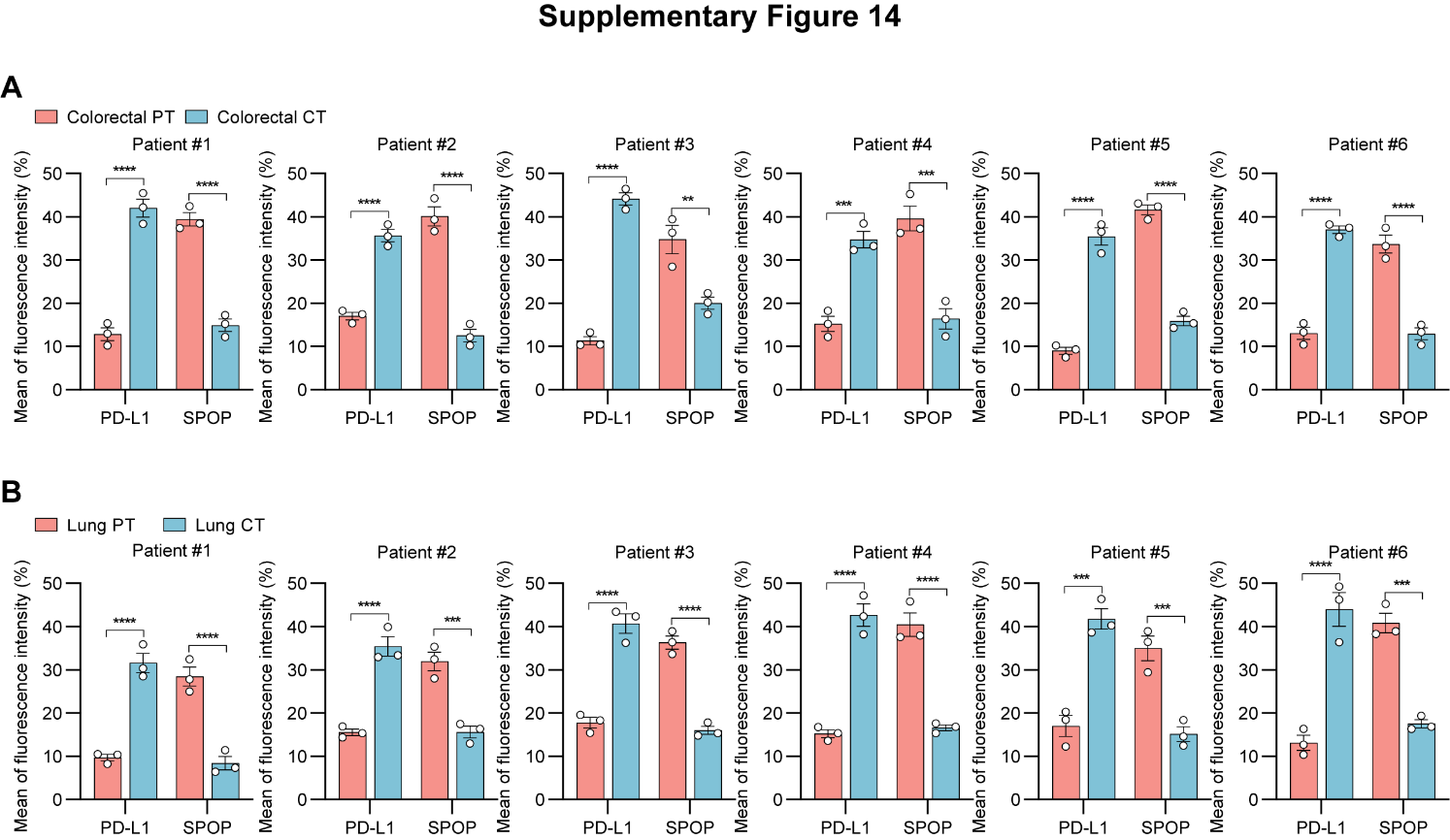


**Figure S11. Clinical relevance of SPOP in colon and lung cancer. (A and B)** (A) and (B) are the quantification results of Figure 8M and Figure 8N, respectively. The data shown are the mean ± standard error of the mean (SEM). Statistical differences were determined by Student’s t test. *p < 0.05; **p < 0.01; ***p < 0.001; ****p < 0.0001; N.S., not significant.
